# Systems evaluation reveals novel transporter YohJK renders 3-hydroxypropionate tolerance in *Escherichia coli*

**DOI:** 10.1038/s41598-020-76120-3

**Published:** 2020-11-04

**Authors:** Thuan Phu Nguyen-Vo, Seyoung Ko, Huichang Ryu, Jung Rae Kim, Donghyuk Kim, Sunghoon Park

**Affiliations:** 1 School of Energy and Chemical Engineering, UNIST, Ulsan, 44919 Republic of Korea; 2 School of Chemical and Biomolecular Engineering, Pusan National University, Busan, 46241 Republic of Korea

**Keywords:** Bacterial systems biology, Bacterial transcription, Bacterial development, Cellular microbiology, Metabolic engineering

## Abstract

Previously, we have reported that 3-hydroxypropionate (3-HP) tolerance in *Escherichia coli* W is improved by deletion of *yieP*, a less-studied transcription factor. Here, through systems analyses along with physiological and functional studies, we suggest that the *yieP* deletion improves 3-HP tolerance by upregulation of *yohJK*, encoding putative 3-HP transporter(s). The tolerance improvement by *yieP* deletion was highly specific to 3-HP, among various C2–C4 organic acids. Mapping of YieP binding sites (ChIP-exo) coupled with transcriptomic profiling (RNA-seq) advocated seven potential genes/operons for further functional analysis. Among them, the *yohJK* operon, encoding for novel transmembrane proteins, was the most responsible for the improved 3-HP tolerance; deletion of *yohJK* reduced 3-HP tolerance regardless of *yieP* deletion, and their subsequent complementation fully restored the tolerance in both the wild-type and *yieP* deletion mutant. When determined by 3-HP-responsive biosensor, a drastic reduction of intracellular 3-HP was observed upon *yieP* deletion or *yohJK* overexpression, suggesting that *yohJK* encodes for novel 3-HP exporter(s).

## Introduction

3-Hydroxypropionic acid (3-HP), a three-carbon carboxylic acid, is an industrially relevant platform chemical. It can be converted to numerous value-added compounds such as acrylate, acrylamide, 1,3-propanediol, and malonic acid^1^. Chemical synthesis methods of 3-HP have been established, but they are costly and involve toxic intermediates^2^. Recently, environmentally-friendly biological methods have been developed, which use various microorganisms as recombinant host, including *Escherichia coli*, *Synechococcus elongatus*, *Pseudomonas denitrificans*, *Klebsiella pneumoniae* and *Saccharomyces cerevisiae*, *Corynebacterium glutamicum*^3–9^. Among them, *E. coli* has been extensively studied due to its rich genomic, physiological, and phenotypic information and availability of well-established genetic tool box^10,11^. However, the growth of *E. coli* strains, even the most tolerant *E. coli* W, is severely inhibited by 3-HP at > 300 mM, a level far lower than the industrially desirable 1.0 M^12,13^.

Toxic effect of short-chain aliphatic acids like 3-HP appears mostly upon their entry into the cytoplasm and/or intracellular production (Supplementary Fig. S1). Once entering or being produced inside the cell, acid molecules dissociate into protons (H^+^) and anions owing to the near-neutral pH of cytoplasm and its high salt concentration^14^. Complicated toxic effects caused by H^+^, anions and/or reaction intermediates of the acid do appear inside cell (Supplementary Fig. S1). Cellular responses to acids and/or low medium pH have been extensively studied in enterobacteria, particularly *E. coli*. These responses can be divided into ‘general’ and ‘specific’ acid-resistance, respectively. Altering of cell-membrane components or its structure, removing H^+^, restoring anion imbalance, etc. can be considered as ‘general’ responses, whereas anions-specific counteractions such as acceleration of cellular metabolisms to remove specific anions and their metabolites into non-toxic products can be classified as ‘specific’ responses^8,15–17^. In the general responses, up-regulation of the proton-consuming mechanism, such as glutamic-, arginine-, lysine-, and/or ornithine-dependent acid resistance, has been well documented^15,18–20^. Up-regulation of SOS responses and chaperons expression have also been well studied^21,22^. However, the ‘anion-specific’ acid-resistant mechanisms have been much less studied. In the case of 3-HP, mutation of *SFA1*, which encodes *S*-(hydroxymethyl)glutathione dehydrogenase in the yeast *Saccharomyces cerevisiae*, is known to reduce the toxic effect of 3-HP, because the mutation improves removal of the highly reactive intermediate 3-hydroxypropionaldehyde (3-HPA)^8^. In the same context, the up-regulation of *frmB*, the *SFA1* ortholog, has also been noted when *E. coli* is growing in the presence of 3-HP^23^.

There have been several attempts to improve tolerance against organic acids. For example, for improved 3-HP tolerance in *E. coli*, a complex nitrogen source like yeast extract, mixture of several amino acids including methionine, threonine, and aromatic amino acids, and/or their biosynthetic intermediates, was added into the culture medium^24^. Up-regulation of biosynthetic pathways for several amino acids has also known to improve tolerance against 3-HP^24^. The so-called ‘adaptive laboratory evolution’ (ALE), repeated exposure of a microorganism to a target acid with an increasing concentration and selecting the fast-growing cell(s), is also a routine and popular practice^8,25,26^. In a recent study, by ALE, we isolated a highly 3-HP-tolerant *E. coli* W strain^27^. The strain contained 13 point-mutations, including alterations in *glpK* and *yieP*, and could grow in the presence of 800 mM 3-HP when yeast extract was supplemented to the culture medium. Furthermore, the strain could produce more 3-HP than its parent counterpart when the mutated *glpK* gene was reverted to the native form. Interestingly, the mutation in one specific gene named *yieP*, encoding a less-characterized transcriptional regulator^28^, was entirely responsible for the improved 3-HP tolerance. When the *yieP* gene was deleted, both the wild-type and the evolved strains exhibited the same tolerance to 3-HP; and when the native *yieP* was re-expressed, both the strains lost their tolerance. Although the transcriptional binding-sites of YieP was reported^28^, the cellular mechanism how the *yieP* deletion confers 3-HP tolerance has not been elucidated.

This study aims to investigate the 3-HP tolerance mechanism acquired by deletion of the global regulator encoded by *yieP*. Specificity of 3-HP tolerance by Δ*yieP*, genome-wide transcription profiles in response to the *yieP* deletion, and systems-level YieP binding landscape were investigated to select and identify genes/operons responsible for the improved 3-HP tolerance. Deletion and subsequent complementation of the selected targets were also conducted to confirm their physiological roles. This study suggests the presence of putative 3-HP exporter(s) encoded by *yohJK*, the upregulation of which is resulted by Δ*yieP* and renders 3-HP tolerance in *E. coli*.

## Results

### Tolerance improvement by *yieP* deletion is highly specific to 3-HP

**Figure 1:**
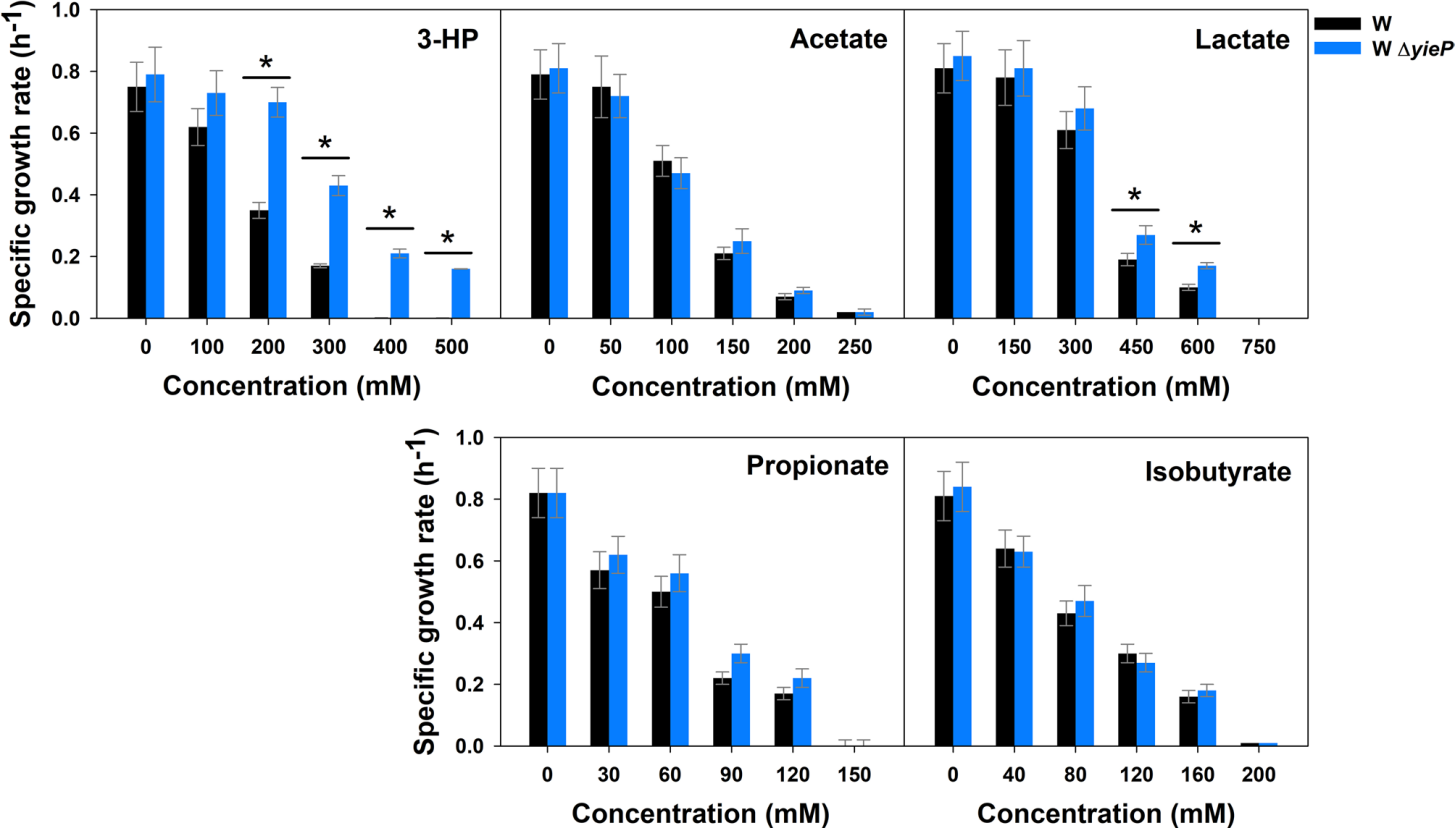
Inhibition of microbial growth by various organic acids. *E. coli* W and its Δ*yieP* mutant were grown in the M9 minimal medium with different organic acids: 3-HP, acetate, lactate, propionate, and isobutyrate. The concentration of each organic acid was varied within the specific respective ranges. Symbols: wild-type *E. coli* W (black bar), *E. coli* W Δ*yieP* (blue bar). Asterisk (*) indicates the significant difference (*p* < 0.05).

To determine the specificity of acid-tolerance improvement by *yieP* knock-out (i.e., whether the improvement is limited to 3-HP only or extended to other organic acids), wild-type *E. coli* W and its Δ*yieP* mutant were grown in the presence of C2–C4 weak organic acids (Fig. 1). Because the minimal inhibitory concentration (MIC) at which cell growth stops is different for each acid^29,30^, the range of acid concentration tested was varied as follows: 1–500 mM for 3-HP, 0–250 mM for acetate, 0–750 mM for lactate, 0–150 mM for propionate, and 0–200 mM for isobutyrate. For 3-HP, it has been confirmed that *yieP* deletion effectively improves tolerance^27^; the *yieP* deletion mutant could grow up to 500 mM, while the wild-type counterpart stopped its growth at < 400 mM. On the other hand, for the rest of the C2–C4 acids, the *yieP* deletion showed only a marginal or negligible improvement of tolerance. In another experiment, we added yeast extract (YE) at 0.5 g/L to the culture medium to examine whether 3-HP tolerance conferred by *yieP* deletion is masked by YE (Supplementary Fig. S2). Yeast extract significantly improved the tolerance in both the wild-type and Δ*yieP* mutant strains. However, the Δ*yieP* mutant still grew better than the wild-type in the presence of 3-HP, and the difference became more significant as the 3-HP concentration increased. In summary, the deletion of *yieP* increased the tolerance specifically against 3-HP, regardless of the presence of yeast extract in the culture medium.

### Transcriptome analysis reveals possible 3-HP tolerance mechanisms

To identify the genes and operons affected by the *yieP* deletion and to obtain insight into the possible molecular mechanisms behind the improved 3-HP tolerance, genome-wide transcriptomic analyses were performed for both the wild-type and Δ*yieP* mutant of *E. coli* W during the growth in M9 minimal medium (Supplementary Fig. S3). The effect of *yieP* deletion and 3-HP addition was elucidated by comparing the gene expression profiles among WΔ( +), W( +), WΔ(−) and W(−) (note that WΔ and W represent the mutant *E. coli* W Δ*yieP* and wild-type *E. coli* W, respectively; ‘ + ’ and ‘−’ in the parentheses indicate the presence and absence of 100 mM of 3-HP in the culture medium, respectively). When cells were cultured in the absence of 3-HP, expression of 279 genes (5.3% of total genes) was significantly altered by the *yieP* deletion (log_2_(fold change) > 1 and *p* value < 0.05). The number of alterations was 1.5-times higher when 3-HP was present in the culture medium (411 genes, 7.8% of total genes). Comparison between W( +) and W(−), showed differential expression in a total of 676 genes (13.0%), out of 5195 genes; among which, 274 genes (5.3%) were up-regulated while 402 genes (7.1%) were down-regulated (Supplementary Fig. S3). On the other hand, the comparison between WΔ( +) and WΔ(−) showed differential expression in a total of 442 genes (8.4%); 215 genes (4.1%) were up-regulated, while 227 genes (4.4%) were down-regulated (Supplementary Fig. S3). Hierarchical clustering of differentially expressed genes from the RNA-seq revealed that the transcriptomic variation by removal of *yieP* was more significant than that by 3-HP supplementation (Supplementary Fig. S3).

**Figure 2:**
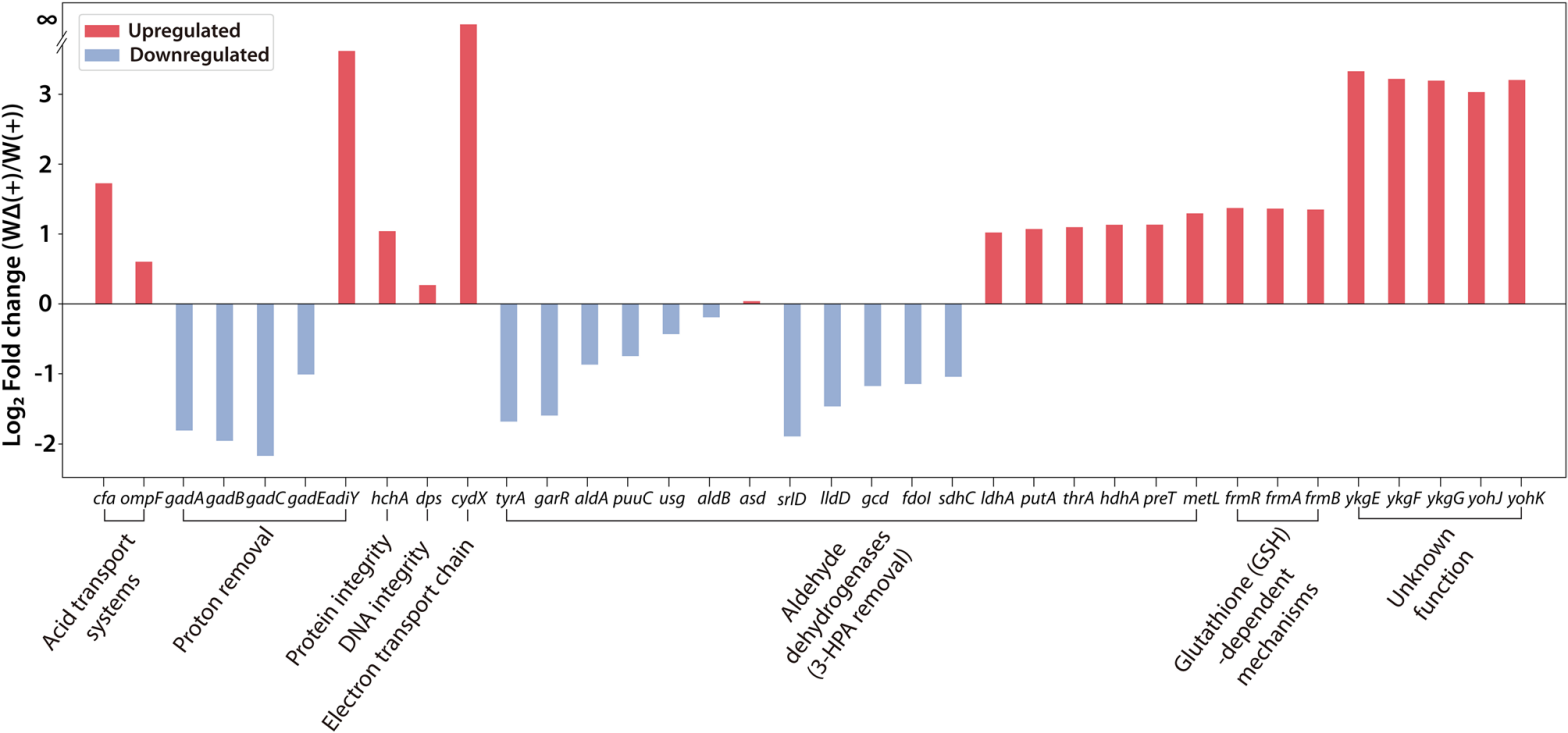
Differentially expressed genes upon *yieP* knock-out. Genes for several ‘general’ acid-resistance responses and/or unknown functions were differentially expressed as consequence of the *yieP* deletion*.* Red and blue indicate up-regulation and down-regulation by *yieP* deletion, respectively. The Y-axis is the log_2_(fold change) between Δ*yieP* and the wild-type in the presence of 3-HP, and the X-axis indicates the gene names and their functions.

Although the *yieP* deletion is highly specific to 3-HP, the ‘general’ acid-resistance mechanisms should appear because 3-HP is an acid. Additionally, the general acid-resistance mechanisms should contribute to 3-HP tolerance to a certain extent in the *yieP* deletion mutant. Therefore, genes known to be related with the general acid-resistance responses were analyzed in the following four categories: (1) acid transport (reduction of influx and activation of efflux); (2) proton removal; (3) maintenance of integrity/activity of proteins, and nucleic acids; and, (4) electron transport chain (Fig. 2 and Supplementary Fig. S4). In the *yieP* deletion strain, the expression levels of *cfa* (cyclopropane fatty acyl phospholipid synthase) and *ompF* (outer-membrane porin F) were up-regulated upon 3-HP addition. On the other hand and contrary to our expectation, the primary glutamate-dependent acid-resistant (GDAR) system, including transcription regulator *gadE* and its regulon genes *gadA* and *gadBC*, showed reduced RNA levels in the *yieP* deletion strain. However, the transcription factor *adiY* for the arginine-dependent response system (ADAR) was significantly up-regulated (12.3-fold). The *hchA* gene encoding the Hsp31 molecular chaperone for protein stabilization was also significantly up-regulated in Δ*yieP* mutant. A similar up-regulation was also observed for *cydX* which encodes for cytochrome bd-I ubiquinol oxidase subunit, a gene involved in the electron transport chain. In summary, many genes responsible for ‘general’ acid-resistant mechanism were up-regulated by *yieP* deletion, except GDAR genes which were down-regulated.

For the ‘3-HP-specific’ or ‘anion-specific’ resistance mechanisms^31^, expression of aldehyde dehydrogenases and glutathione (GSH)-dependent dehydrogenases was examined (Fig. 2). In the Δ*yieP* mutant, among a total of 18 genes encoding (putative) aldehyde dehydrogenases, 7 genes were up-regulated whereas 11 other genes were down-regulated. It is noted that the *frmRAB* operon, known to be associated with a glutathione (GSH)-dependent detoxification mechanism and reported to be up-regulated by 3-HP in a previous study^23^, was up-regulated by 2.90-fold by Δ*yieP*. To identify other possible ‘3-HP-specific’ resistance mechanisms, the genes with unknown functions but showing highly differential expression were further screened and scrutinized. Two operons, *ykgEFG* and *yohJK*, drew our attention because of a significant up-regulation (~ tenfold) by the *yieP* deletion. The *yieP* gene is a less-characterized transcription factor; thus, functionally-unknown genes can be highly relevant.

### ChIP-exo enables systems-level reconstruction of YieP regulatory network

RNA-seq analysis identified the genes and operons that were affected by 3-HP and *yieP* deletion, but they were too many for functional studies. Another useful genome-wide tool for identification of genes/operons regulated by a transcription factor such as YieP, is the ChIP-exo analysis^32,33^. According to a recent ChIP-exo study on *E. coli* K-12 MG1655, there exist ~ 23 binding sites for YieP^28^. Here, we attempted to analyze YieP regulon in *E. coli* W by comparing ChIP-exo data and RNA-seq data. Note that the ChIP-exo data for *E. coli* MG1655 was used instead of that for *E. coli* W. The reason for that is because *E. coli* K-12 MG1655 has the most comprehensive knowledge on the transcriptional regulatory network, and ChIP-exo experimental protocol, ready to be used, was set up for that particular strain. On the other hand, genomic comparison showed that these two strains shared over 84.1% of the genes (Supplementary Fig. S5) and, more importantly, they had the same sequences for all the YieP target sites.

**Figure 3:**
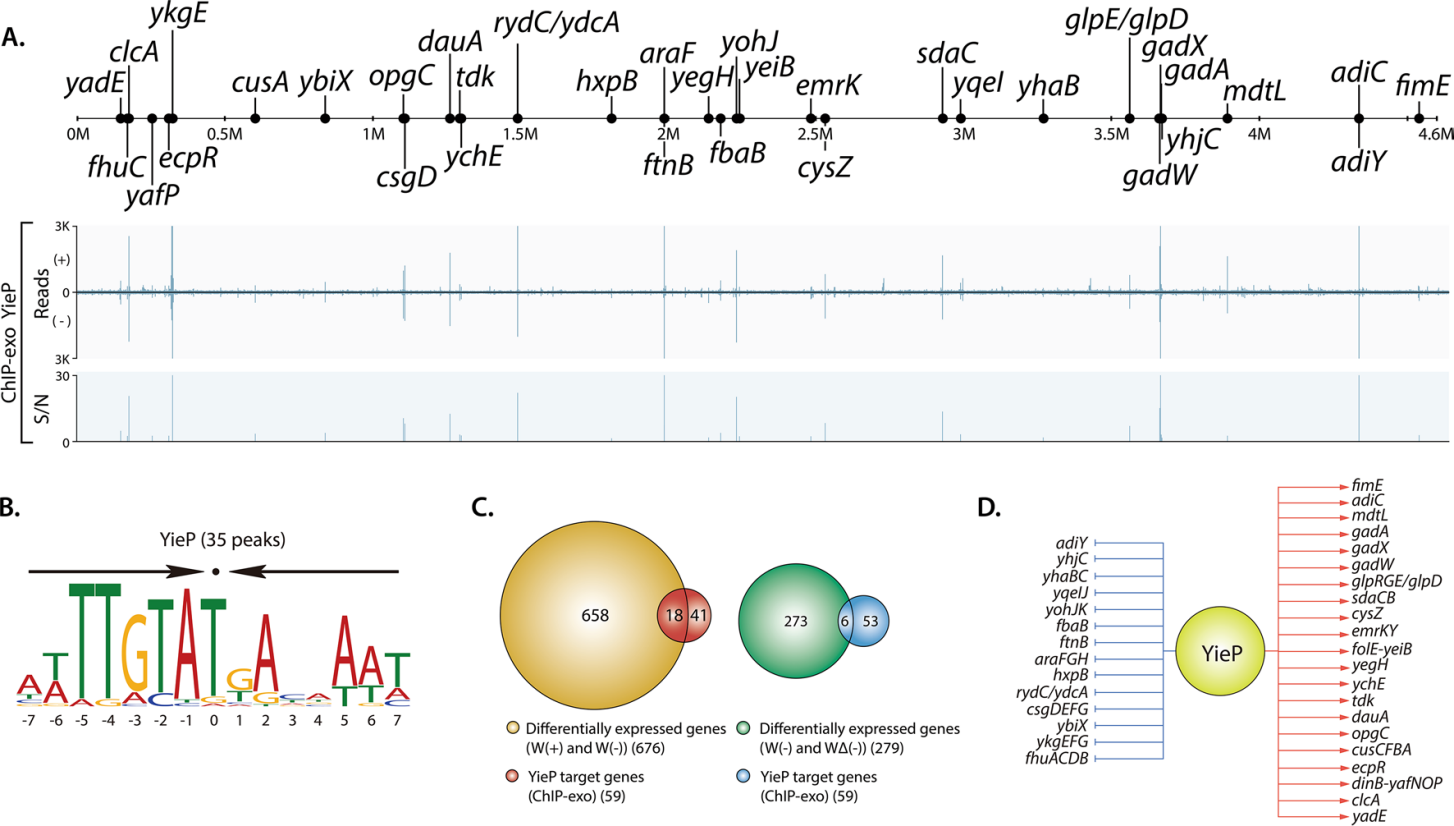
Genome-wide identification of YieP regulon. (**A**) An overview of YieP-binding profiles across the genome of *E. coli* K-12 MG1655 cultured in the presence of 3-HP. (**B**) The sequence motif for YieP transcription factor. Arrows above the motif indicate the palindromic sequence. (**C**) Comparison of ChIP-exo result and gene expression profile to distinguish direct and indirect regulatory effects for YieP regulon genes. (**D**) Reconstruction of YieP regulon genes. The red and blue arrows indicate activation and suppression, respectively.

The ChIP-exo analysis for *E. coli* K-12 MG1655 with the addition of 3-HP revealed 33 YieP binding sites on the genome-scale (Fig. 3A). Among them, 12 binding sites were located in the intragenic regions, and 21 were in the intergenic regions. Integration of the identified binding sites by ChIP-exo, transcriptomic profiling by RNA-seq and operon structures known thus far^34,35^ revealed the presence of 33 transcriptional units (TUs) regulated by YieP, which contain a total of 59 genes. A sequence-motif analysis of the YieP binding sites showed the presence of a palindromic sequence: atTTGTaTGAcaAAT (capital characters indicating information content > 1 bit) (Fig. 3B). This palindromic sequence motif suggests that YieP might work as a dimeric conformation. Among the 59 genes belonging to the 33 YieP regulons, a total of 33 target genes were up-regulated, and the remaining 26 were down-regulated (Fig. 3D). Notably, six genes (*ykgEFG*, *yohJK*, and *adiY*) in 3 TUs were down-regulated by YieP with high-log_2_(fold change) as: < 4.8, 5.4, and 2.2-fold, respectively. This regulatory mode of YieP is consistent with the previous study^28^.

To minimize and finalize the list of genes for detailed functional studies, the 676 target genes suggested by the RNA-seq and 59 target genes indicated by the ChIP-exo analysis, respectively, were further curated. Five categories, individually or in combinations, were looked into for this purpose, as follows (Supplementary Fig. S6): (1) difference between *E. coli* W and *E. coli* BL21(DE3) (notice that, according to our previous study^27^, BL21(DE3) did not show tolerance improvement by the *yieP* deletion), (2) high-fold change in gene expression caused by the *yieP* deletion, (3) general acid-resistance mechanisms, (4) genes for membrane synthesis, and (5) genes with unknown functions. For the first category, the DNA sequences of TUs which are presumably under the control of YieP in *E. coli* BL21(DE3) and *E. coli* W or *E. coli* K-12 MG1655 were compared, and the 2 operons, *ykgEFG*, and *sdaCB*, were chosen due to the difference in their promoter sequences (Supplementary Fig. S7). In the second category, for the highly up-regulated (~ tenfold) genes resulted by the *yieP* deletion, three operons, *ykgEFG*, *yohJK* and *adiY*, were chosen. In the third category, for the general acid-resistant genes, *gadX* and *adiY*, which are known to be complicated acid-resistant regulatory proteins, were selected^15,18^. In the fourth category, the *rydC* gene, encoding a small RNA and known to be related to the biosynthesis of putative transporters and curli, was chosen^36^. Finally, in the fifth category, the *ydcA* gene, the function of which is unknown, was selected. The *ykgEFG* and *yohJK* can also be classified as the fifth category, although chosen in the second category. Altogether, a total of 11 genes in 7 operons were selected as prospective candidates for further investigation.

### Transcription of YieP target genes varies in multiple *E. coli* strains

**Figure 4:**
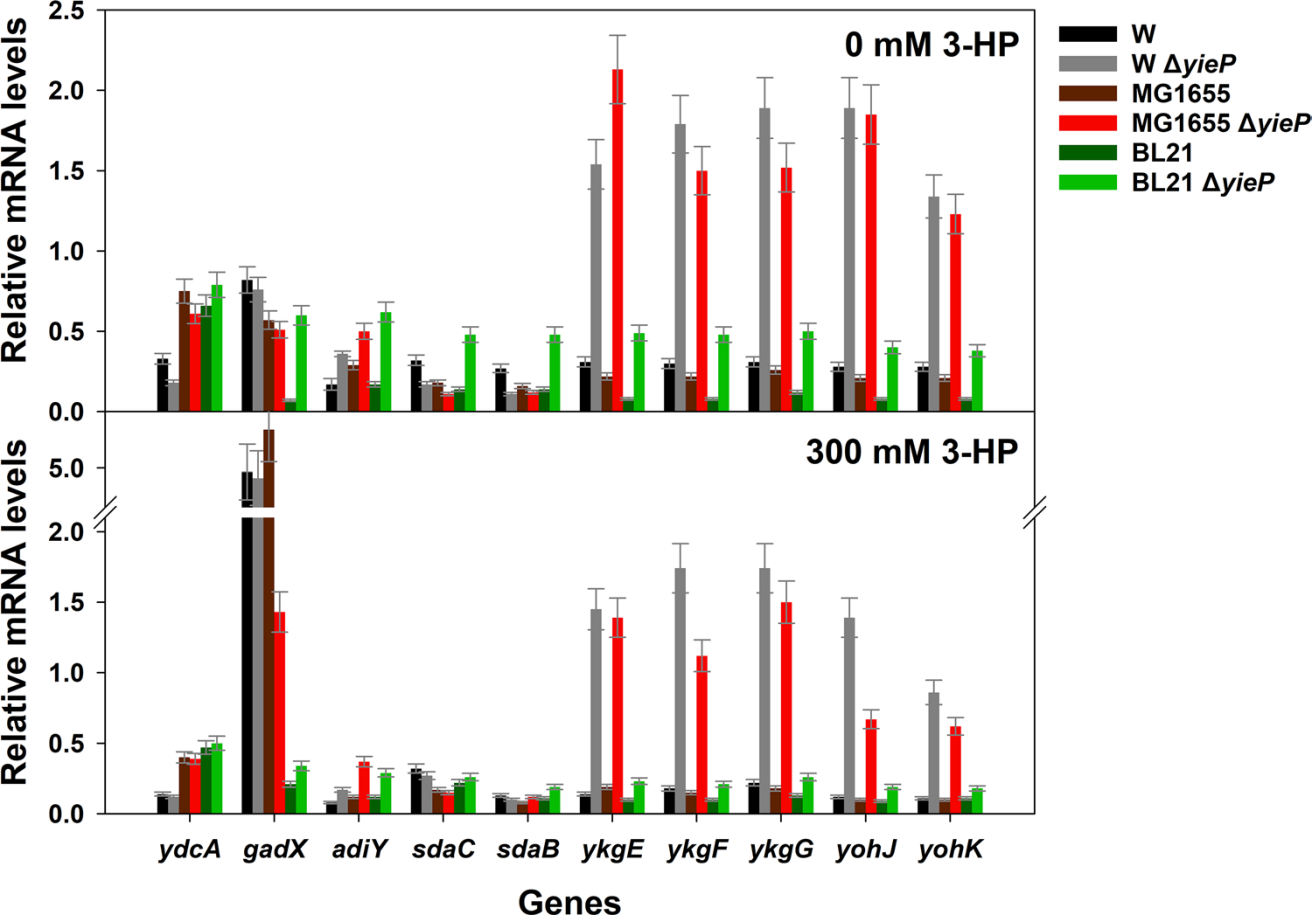
Relative mRNA transcription levels of 10 selected genes. Transcription was determined in *E. coli* W, *E. coli* K-12 MG1655, *E. coli* BL21(DE3), and their corresponding Δ*yieP* mutants grown with and without 300 mM 3-HP (**upper** and **lower** panel, respectively). The housekeeping sigma factor, *rpoD*, was used as reference. Symbols: *E. coli* W (black bar), *E. coli* W Δ*yieP* (gray bar), *E. coli* K-12 MG1655 (dark-brown bar), *E. coli* K-12 MG1655 Δ*yieP* (red bar), *E. coli* BL21 (DE3) (dark-green bar), *E. coli* BL21(DE3) Δ*yieP* (light-green bar).

Knock-out of *yieP* improved 3-HP tolerance in several *E. coli* strains, including *E. coli* W, *E. coli* K-12 MG1655, *E. coli* K-12 W3110, and *E. coli* B, but not in *E. coli* BL21(DE3)^27^. It was postulated that the phenotypic difference of *E. coli* BL21(DE3) might be originated from differential gene expression of the YieP regulons. For clarification of this hypothesis, functional analyses of the 11 target genes with their deletion and over-expression are needed, which is time-consuming and laborious. Therefore, before the functional analyses, the selected target genes were examined by RT-qPCR experiments in the three *E. coli* strains, W, K-12 MG1655, and BL21(DE3), and their three corresponding Δ*yieP* mutants, respectively. Ten genes among the 11, except for *rydC* the length of which is too short (64 bp), were analyzed (Fig. 4). For *E. coli* W, RT-PCR analysis showed a good correlation with FPKM values from the RNA-seq analysis (correlation coefficient was 0.71) (Supplementary Fig. S8). Among the 10 target genes, the two operons *ykgEFG* and *yohJK* exhibited a highly enhanced transcription by *yieP* deletion, regardless of 3-HP. However, in *E. coli* BL21(DE3), the fold change of these two operons was much lower (~ 2.5-fold) than in the other *E. coli* strains (~ 6 to 10-fold). The *adiY* gene, a transcription regulator for the arginine-dependent acid-resistant (ADAR) system, was up-regulated by ~ twofold in all of the Δ*yieP* mutants, unlike the corresponding wild-type strains. Expression of the regulator related to the glutamate-dependent acid-resistant system, *gadX*, was not affected by the *yieP* deletion, but the addition of 3-HP to the culture medium up-regulated its transcription level as high as sixfold in *E. coli* W and *E. coli* K-12 MG1655. Transcription of the remaining genes (*ydcA* and *sdaCB*) was not affected by the *yieP* deletion. These results expand our knowledge on the regulatory role of *yieP* from *E. coli* W to other *E. coli* strains, especially by demonstrating that YieP down-regulates expression of multiple genes, including *ykgEFG*, *yohJK*, and *adiY*. These genes are expected to play important roles in linking Δ*yieP* genotype to 3-HP tolerant phenotype.

### *yohJK* operon, a member of the YieP regulon, is essential for ‘3-HP-specific’ tolerance

**Figure 5:**
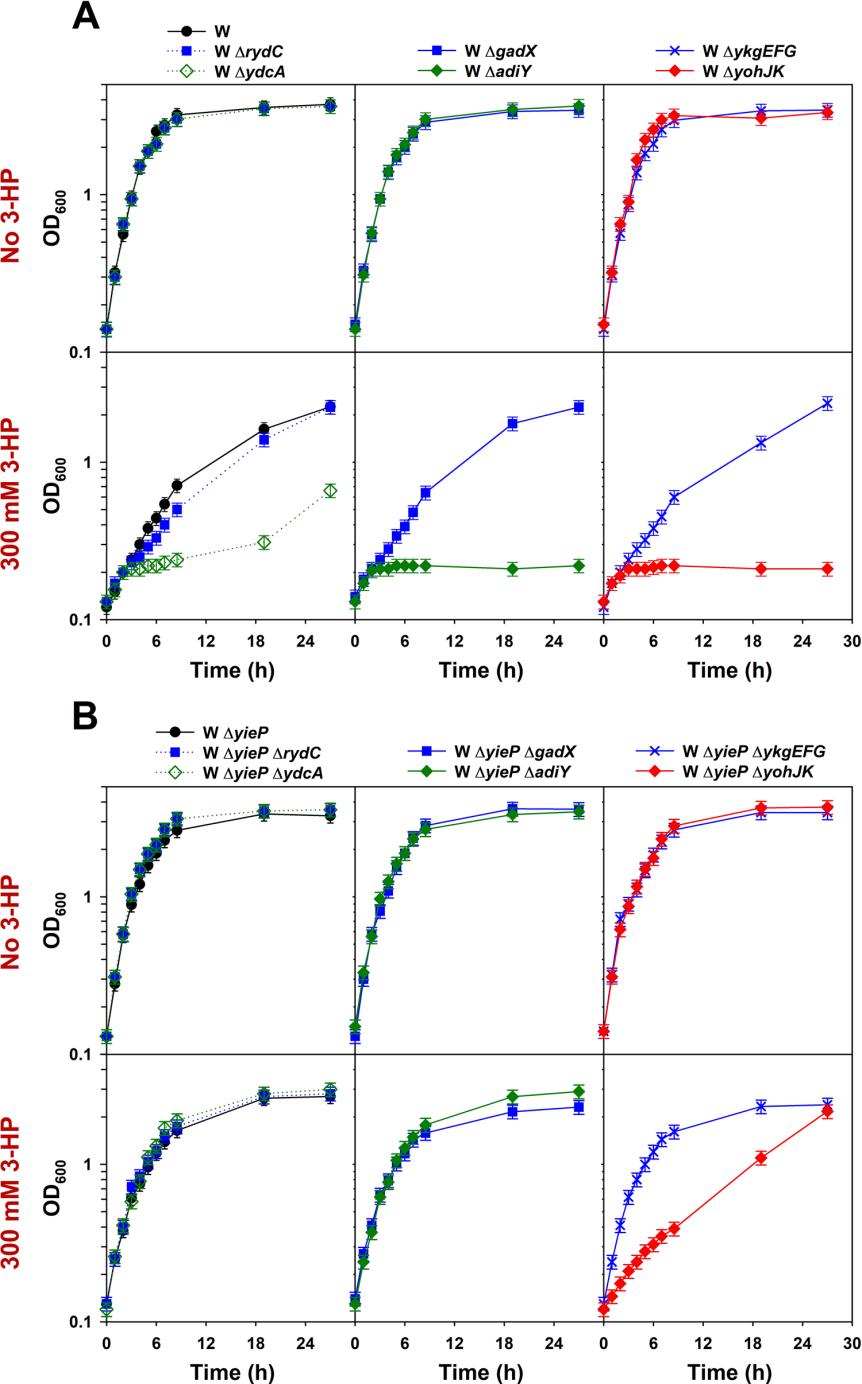
Identification of genes/operons conferring 3-HP tolerance. Six selected genes/operons were individually deleted from *E. coli* W (**A**) and *E. coli* W Δ*yieP* (**B**). All the mutants were grown in the modified M9 minimal medium. The 300 mM 3-HP was supplemented to the culture medium when indicated. Symbols: *E. coli* W (wild-type and Δ*yieP*; black circles, solid line), Δ*rydC* (blue closed rectangles, dotted line), Δ*ydcA* (green open diamonds, dotted line), Δ*gadX* (blue closed rectangles, solid line), Δ*adiY* (green closed diamonds, solid line), Δ*ykgEFG* (blue crosses, solid line), and Δ*yohJK* (red closed diamonds, solid line).

In order to evaluate functional importance, six operons (*rydC*, *ydcA*, *gadX*, *adiY*, *ykgEFG*, and *yohJK*, except *sdaCB*) were subjected to deletion experiments. Two series of deletion mutants were developed: the first from the wild-type *E. coli* W, and the second from its Δ*yieP* mutant, respectively (Supplementary Table S1). The growth of these mutants (12 strains in total) was examined in the modified M9 minimal medium (Fig. 5A,B). When 3-HP was absent, the mutations did not disturb cell growth for either the wild-type (for *yieP*) strains or their Δ*yieP* mutants. However, when 3-HP (300 mM) was added, the growth of the three mutants in the first series (*ydcA*, *adiY*, and *yohJK*), developed from the wild-type *E. coli* W, was severely reduced (Fig. 5A). On the other hand, in the second series of mutants, those developed from the Δ*yieP* mutant, 3-HP toxic effect was much reduced: entirely in the case of the *ydcA* and *adiY* mutants, and partially in the case of the *yohJK* mutant (Fig. 5B).

**Figure 6:**
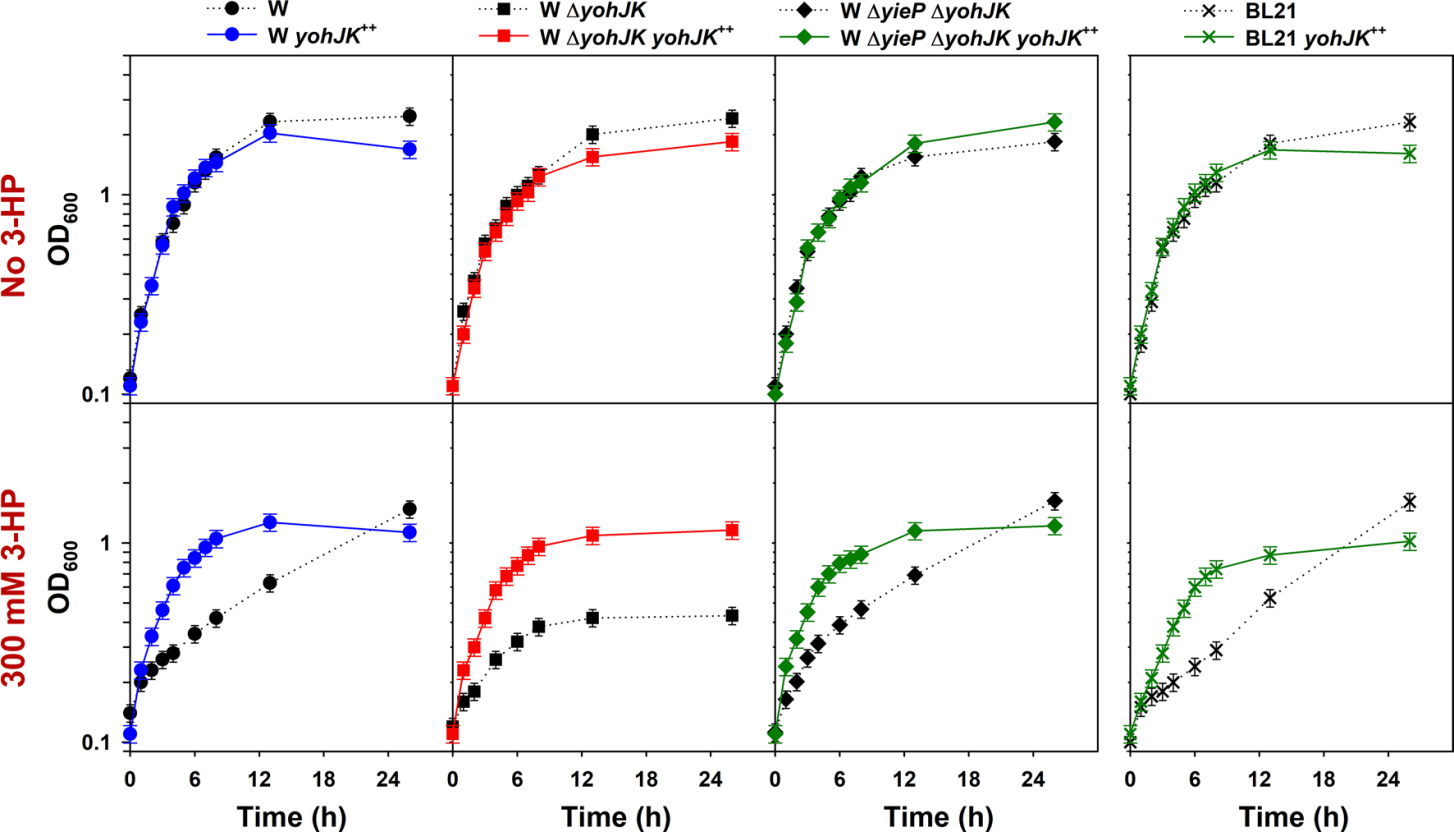
Overexpression of *yohJK* in *E. coli* strains. Recombinant *E. coli* strains were grown in the M9 minimal medium containing 50 mg/L kanamycin. 3-HP was added to the culture medium when indicated. “*yohJK*^++^” indicates the recombinant strains harboring pACYC-*P*_*tet*_-*yohJK*; otherwise, strains harbor the pACYC empty-plasmid. Symbols: *E. coli* W (black circles, dotted line), *E. coli* W *yohJK*^++^ (blue circles, solid line), *E. coli* W Δ*yohJK* (black rectangles, dotted line), *E. coli* W Δ*yohJK yohJK*^++^ (red rectangles, solid line), *E. coli* W Δ*yieP* Δ*yohJK* (black diamonds, dotted line), *E. coli* W Δ*yieP* Δ*yohJK yohJK*^++^ (green diamonds, solid line), *E. coli* BL21(DE3) (black crosses, dotted line), *E. coli* BL21(DE3) *yohJK*^++^ (green crosses, solid line).

Because the *yohJK* deletion overrides the *yieP* deletion in showing 3-HP tolerance, overexpression of *yohJK* on 3-HP tolerance was also investigated (Fig. 6). The *yohJK* genes were cloned into the low-copy pACYC plasmid under the tetracycline-inducible promoter (*P*_*tet*_). The expression of *yohJK* was induced (by adding 200 ng/mL of anhydrotetracycline) for one hour before adding 300 mM 3-HP. With overexpression of *yohJK* (designated *yohJK*^++^), the wild-type *E. coli* W exhibited an improved tolerance to 3-HP (Fig. 6). This indicates that the overexpression of *yohJK* alone, without the *yieP* deletion, can confer 3-HP tolerance to *E. coli* W. However, the tolerance level in the *yieP* deletion mutant was not further enhanced by overexpression of *yohJK* (Supplementary Fig. S10). The role of *yohJK* was also confirmed by complementary expression of *yohJK* in both *E. coli* W Δ*yohJK* and *E. coli* W Δ*yieP*Δ*yohJK*: both the strains exhibited similar tolerance to 3-HP as *E. coli* W *yohJK*^++^. Interestingly enough, over-expression of *yohJK* improved the 3-HP tolerance in *E. coli* BL21(DE3), which did not show improvement at all by the deletion of *yieP*. It is likely that the lower up-regulation of *yohJK* in *E. coli* BL21(DE3) (~ 2.5 fold) than in other *E. coli* strains (~ 6 to 10-fold) is responsible for not acquiring 3-HP tolerance by the *yieP* deletion in the former strain. It will be interesting to overexpress *yohJK* in other microbes in order to improve their tolerance to 3-HP.

### *yohJK* operon encodes for 3-HP transporter in *E. coli* W

The results of this study thus far strongly suggest that the improvement of 3-HP tolerance caused by the *yieP* deletion is mostly mediated by the up-regulation of *yohJK*. However, the exact mechanism behind the tolerance improvement remains elusive. According to EcoCyc, *yohJ* and *yohK* are predicted to be inner-membrane proteins with four and six transmembrane domains, respectively^37,38^, suggesting that the protein(s) are transporter(s) for 3-HP. To study their physiological roles, we measured intracellular 3-HP concentration by 3-HP responsive biosensor. The biosensor module employs a LysR-type transcription factor (MmsR) which is specifically activated by 3-HP and induces the expression of green fluorescence protein (GFP) under the control of P_mmsA_ promoter^39^ (Supplementary Fig. S11 and S12). Because the one (BS-P) previously reported was not sensitive in *E. coli*, a new biosensor module (BS-E) having a higher expression of MmsR was constructed (see Materials and Method). When tested in the modified M9 minimal medium, the strain with the new BS-E module gave ~ threefold higher GFP signal than the one with its predecessor BS-P. Both BS-P and BS-E showed increased GFP intensity up to 25 mM 3-HP in a dose-dependent manner.

**Figure 7:**
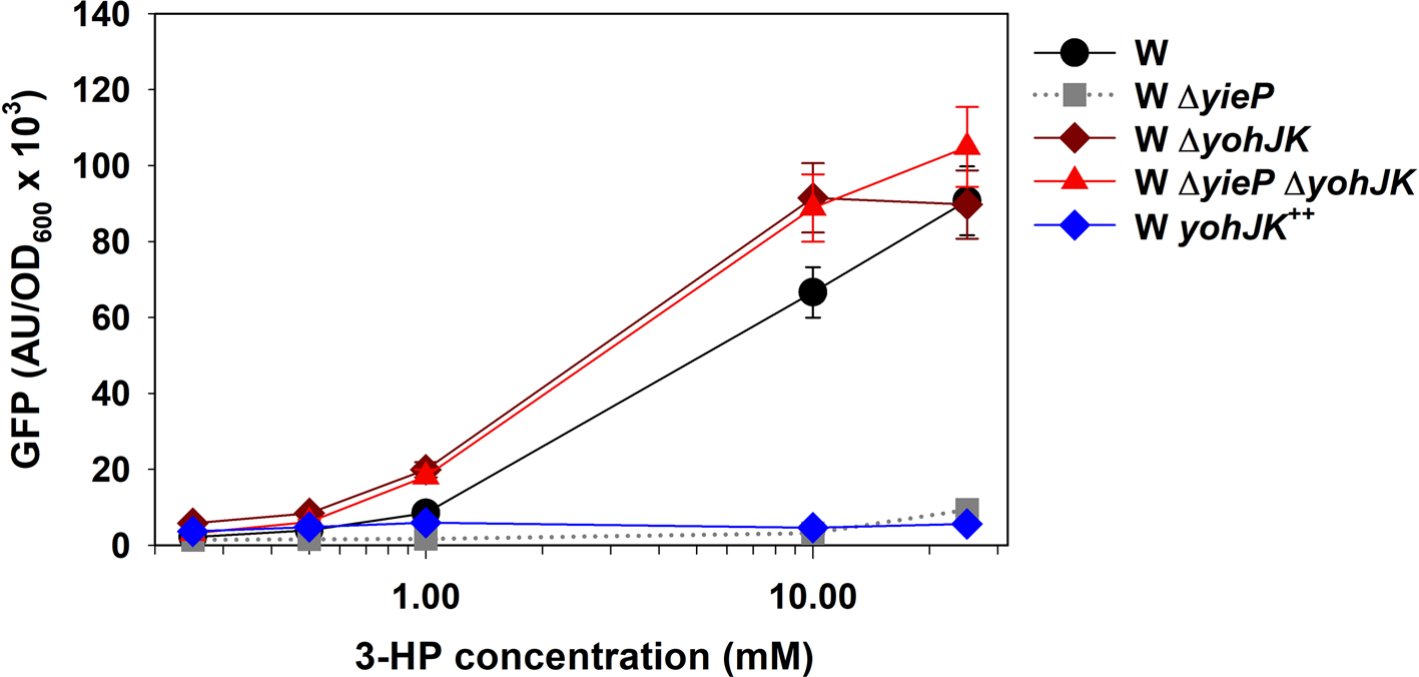
Effect of Δ*yieP* , Δ*yohJK* and *yohJK*^++^ on GFP-intensity of 3-HP biosensors. Five *E. coli* W strains (W, W Δ*yieP*, W Δ*yohJK*, W Δ*yieP*Δ*yohJK*, and W *yohJK*^++^) harboring the BS-E biosensor module were grown in the modified M9 minimal medium containing varying 3-HP concentration (0–25 mM). GFP was measured 6 h after inoculation. Symbols: *E. coli* W (black circles); W Δ*yieP* (gray rectangles); W Δ*yohJK* (dark-red diamonds); W Δ*yieP*Δ*yohJK* (red triangles); W *yohJK*^++^ (blue diamonds).

The new biosensor module (BS-E) was introduced into five *E. coli* W strains such as wild type, two single deletion mutants for each of *yieP* and *yohJK*, a double deletion mutant for both *yieP* and *yohJK*, and an *yohJK*-overexpressed strain (*yohJK*^++^), and the GFP signals were compared among the five strains in varying 3-HP concentrations (from 0 to 25 mM). The GFP intensity was determined 6 h after inoculation (Fig. 7). The intensity increased in the following order (notably, up to 10 mM 3-HP): *yohJK*^++^  ≤ Δ*yieP* < W (wild-type) <  Δ*yieP*Δ*yohJK* ≤ Δ*yohJK.* It is noticed that the *yieP* deletion mutant (where *yohJK* is upregulated according to RT-PCR shown in Fig. 4) and the *yohJK*-overexpressed strain (*yohJK*^++^), showed a dramatically reduced GFP intensity compared to that of the wild type. On the other hand, the *yohJK* deletion in both wild-type and Δ*yieP* mutant substantially increased the GFP signal. These results indicate that the intracellular 3-HP concentration is significantly reduced when *yieP* is deleted or *yohJK* is overexpressed, and support our hypothesis that the physiological function of the *yohJK* genes is involved with the excretion or removal of 3-HP from the cell. Because *yohJK* encode for integral transmembrane proteins and their overexpression reduces intracellular 3-HP concentration greatly, the protein(s) is likely a novel 3-HP exporter.

## Discussion

We wanted to study the mechanism how the *yieP* deletion improves 3-HP tolerance and use the information to develop better 3-HP tolerant strains. Without knowing the physiological functions of YieP, the first thing that should be done was screening the candidate genes and operons affected by YieP or its deletion. Although YieP was suggested as a transcription factor^28^, the possibility to conduct other physiological functions was not fully eliminated a priori. The specificity experiment with different organic acids was conducted as the first step to search the uncovered mechanism(s), and it was found that the effect of the *yieP* deletion was highly specific to 3-HP. Consequently, the ‘3-HP specific’ mechanism was targeted for further investigations (Fig. 1).

The combination of transcriptomics, mapping of transcriptional binding sites, genomics, and physiological study was an efficient approach for elucidation the role of *yieP* deletion. Genome-wide transcriptomic profiling and ChIP-exo analyses are useful when a global regulator is studied for its function^28^. In this study, these two, genome-wide approaches significantly narrowed down the number of candidates from the initial 411 genes to 59 genes (Fig. 3). Comparison of genomic sequences (Supplementary Fig. S7) and/or transcription levels (Fig. 4) between *E. coli* BL21(DE3), that was insensitive to the *yieP* deletion, and other *E. coli* strains also helped the selection of target genes. Through all these sequential efforts (Supplementary Fig. S6), the number of operons to be studied could be minimized to six (*rydC*, *ydcA*, *gadX*, *adiY*, *ykgEFG*, and *yohJK*) for the functional analyses, i.e., knockout and/or overexpression experiments.

According to functional analyses, the deletion of *ydcA* or *adiY* made cells sensitive to 3-HP, but the sensitivity was overridden by the *yieP* deletion completely (Fig. 5). The positive transcriptional factor, *adiY*, has been suggested to regulate both the arginine- and glutamate-dependent acid-resistant systems^40^. Similarly, the *ydcA* protein has also known to be related with acid-resistance. It was reported that the expression of *ydcA*, under the control of YdcI, was up-regulated in acidic condition (pH 5.0) (Supplementary Fig. S9)^28,41^. However, the involvement of AdiY and YdcA in 3-HP tolerance is not clear. The deletion of *yohJK* also made cells sensitive to 3-HP and, furthermore, the sensitivity was not overridden by the *yieP* deletion, differently from the *ydcA* or *adiY* deletion (Fig. 5). In addition, the overexpression of *yohJK* without *yieP* deletion could improve the 3-HP tolerance as much as the *yieP* deletion (Supplementary Figs. S6 and S10). This indicates the effect of *yieP* deletion on 3-HP tolerance, in a large extent, is mediated by the *yohJK* overexpression. The function of *yohJ* and *yohK* in *E. coli* has not been revealed previously, but according to EcoCyc, *yohJ* and *yohK*, both are inner-membrane proteins with four and six transmembrane domains, respectively^37^. Further study using biosensor revealed that the up-regulation of *yohJK* significantly reduces intracellular 3-HP concentration while their deletion increases its level, strongly suggesting they encodes for 3-HP-specific exporter(s) (Fig. 7). If YohJK is a 3-HP-specific transporter, it is understandable why the *yohJK* overexpression, either indirectly by *yieP* deletion or directly by its episomal expression, has specifically improved 3-HP tolerance. Thus far, no 3-HP-specific transporter has been reported, and further, no functional study on YohJK has been conducted. More studies to understand how YohJK work, i.e., whether both YohJ and K function together or individually, they are associated with other membrane proteins, they utilize energy for 3-HP transport, they can improve 3-HP tolerance in other microorganisms, etc. should be followed.

In conclusion, by combined studies of microbial growth, RNA-seq, ChIP-exo and functional analyses of genes and operons, we could identify novel membrane proteins YohJK which improve 3-HP tolerance in *E. coli* W. YohJK efficiently reduced intracellular 3-HP concentration and should be useful in strain development for achieving high 3-HP titer. In addition, the procedures described in this manuscript should be useful in identifying genes and operons which are affected by a global regulator, the physiological functions of which has not known. Biochemical and physiological aspects of the new putative exporter(s) YohJK and their application to other microbes are under study.

## Methods

### Strains, culture conditions, and materials

*Escherichia coli* W (ATCC 9637) and *E. coli* K-12 MG1655 were obtained from Korean Collection for Type Cultures (KCTC, South Korea). *E. coli* BL21 (DE3) was purchased from Novagen (Darmstadt, Germany). All of the bacterial strains used in this study are listed in Supplementary Table S1. Highly-pure 3-HP sodium salt (> 99%) was provided by Noroo Holdings Ltd, Seoul, Korea. Glycerol, lactic acid and propionic acid were acquired from Sigma-Aldrich (MO, USA). All of the other chemicals were purchased from Junsei, Japan.

Bacterial cells were cultured in modified M9 minimal medium with glycerol as the carbon source, unless indicated otherwise. The modified M9 minimal medium contains: MgSO_4_·7H_2_O, 0.25 g/L; NaCl, 1.0 g/L; NH_4_Cl, 0.5 g/L; yeast extract, 0.5 g/L (optional); potassium phosphate buffer (pH 7.0), 100 mM; and glycerol, 100 mM. Cells were cultured aerobically in a 125 mL flask (working medium, 10 mL) at 37 °C at an agitation speed of 200 rpm^27^. Organic acids were prepared as 10 M stock solutions, neutralized by NaOH and added to the culture medium as needed. To monitor growth, the bacterial cell density (cell OD_600_) was determined at 600 nm by UV/Vis spectrophotometry (Lambda 265, PerkinElmer, CT, USA). All experiments were conducted in duplicate, the results were expressed by means value.

### Genome-wide transcriptome analysis with RNA-seq

For RNA-seq expression profiling, total RNA, including small RNAs, was isolated using the NucleoSpin RNA XS (Macherey–Nagel, Germany). The sequencing library was prepared by random fragmentation of cDNA samples, followed by 5′ and 3′ adapter ligation using TrueSeq stranded mRNA kits (Microbe). Sequencing was performed on a Hiseq 4000 sequencer obtained from Marcrogen Inc. (Korea). RNA-seq experiments were performed in biological duplicate.

For the processing of RNA-seq output, sequence reads generated from RNA-seq were mapped onto the reference genome (NC_017635.1) using Bowtie with the maximum insert size of 1000 bp along with two maximum mismatches after trimming 3 bp at 3′ ends^42^. SAM files generated from Bowtie were then used for Cufflinks (https://cufflinks.cbcb.umd.edu) and Cuffdiff to calculate fragments per kilobase of exon per million fragments (FPKM) and differential expression, respectively. Cufflinks and Cuffdiff were run using default options with the library type of dUTP RNA-seq by the default normalization method (classic-fpkm). From the Cuffdiff output, genes showing differential expression with log2-fold change > 1.0 and a *p* value < 0.05 were considered as differentially expressed genes. Genome-scale data were visualized using MetaScope (https://sites.google.com/view/systemskimlab/software).

### Chromatin immunoprecipitation with exonuclease (ChIP-exo) to identify YieP binding sites

In order to execute ChIP-exo experiments, a multi-Myc-tag peptide sequence was added to the C-terminal of *yieP* using the λ red-mediated site-specific recombination system^43^. The resultant recombinant strain, *E. coli* K-12 MG1655 *yieP-8myc*, was cultured in fresh M9 minimal medium (100 mM phosphate buffer pH 7.0, 1 g/L NaCl, 1 g/L NH_4_Cl, 2 mM MgSO_4_) with 100 mM glycerol as the sole carbon source; 1 mL of trace element solution (100X) containing 1 g/L EDTA, 29 mg/L ZnSO_4_·7H_2_O, 198 mg/L MnCl_2_·4H_2_O, 254 mg/L CoCl_2_·6H_2_O, 13.4 mg/L CuCl_2_ and 147 mg/L CaCl_2_ was also supplemented to the culture medium. When the effect of 3-HP was tested, 100 mM 3-HP was added to the culture medium. The cells were cultured at 37 °C and 200 rpm and harvested in the mid-log phase (~ 0.3 OD_600_) for further experimentation.

A ChIP-exo experiment was performed following the procedures previously described^33^. In brief, to identify the in vivo YieP binding map, DNA bound to YieP from formaldehyde-cross-linked *E. coli* cells was isolated by chromatin immunoprecipitation (ChIP) with the specific antibodies that specifically recognize Myc-tag (9E10, Santa Cruz Biotechnology, Texas, USA) and Dynabeads Pan Mouse IgG magnetic beads (Invitrogen, CA, USA), followed by stringent washing as described previously^34^. ChIP materials (chromatin-beads) were used to perform on-bead enzymatic reactions of the ChIP-exo method^33,44^. Briefly, the sheared DNA of the chromatin-beads was repaired by the NEBNext End Repair Module (New England Biolabs, MA, USA) followed by the addition of a single dA overhang and ligation of the first adaptor (5′-phosphorylated) using the dA-Tailing Module (New England Biolabs) and the NEBNext Quick Ligation Module (New England Biolabs), respectively. Nick repair was performed by using PreCR Repair Mix (New England Biolabs). Lambda exonuclease- and RecJf nuclease-treated chromatin were eluted from the beads, and overnight incubation at 65 °C reversed the protein-DNA cross-link. RNAs- and Proteins-removed DNA samples were used to perform primer extension and second adaptor ligation, respectively, with the following modifications. The DNA samples incubated for primer extension, as described previously, were treated with dA-Tailing Module (New England Biolabs) and NEBNext Quick Ligation Module (New England Biolabs) for second adaptor ligation^33^. The DNA sample purified using the GeneRead Size Selection Kit (Qiagen) was enriched by polymerase chain reaction (PCR) using Phusion High-Fidelity DNA Polymerase (New England Biolabs). The amplified DNA samples were purified again with the GeneRead Size Selection Kit (Qiagen, Hilden, Germany) and quantified using the Qubit dsDNA HS Assay Kit (Life Technologies). The quality of the DNA sample was checked by running the Agilent High Sensitivity DNA Kit using Agilent 2100 Bioanalyzer (Agilent, CA, USA). Sequencing was performed on a Hiseq 4000 sequencer at Marcrogen Inc. (Korea). Each modified step was also performed as per the manufacturer’s instructions. The ChIP-exo experiments were performed in biological duplicate.

In order to process the ChIP-exo output, ChIP-exo analysis was performed according to the method reported previously^28^. The sequence reads of ChIP-exo were mapped onto the reference genome (NC_000913.2) using the Bowtie with default options to generate SAM output files. To reduce false-positive peaks, peaks with a signal-to-noise (S/N) ratio < 1.5 were removed. The noise level was set to the top 5% of signals at genomic positions, because the top 5% makes a background level in a plateau, and the top 5% intensities from each of the ChIP-exo replicates across conditions correlate well with the total number of reads. Then, each peak was assigned to the nearest gene. Genome-scale data were visualized using MetaScope.

A YieP-binding motif analysis was performed using the MEME tool from the MEME software suite^45^. YieP-binding DNA sequences were extracted from the reference sequence (NC_000913.2). The sequence of each binding site was extended by 20 bp at each end to allow for adjacent sequences to be included in the analysis. The default setting was used, except for the width parameter, which was fixed to 20 bp.

### Homologous recombination for gene disruption

Mutants with multiple knock-outs were well developed using a method of scarless homologous recombination reported elsewhere^11^. In brief, the upstream and downstream fragments of a target gene were amplified using PCR, and overlapping PCR was performed to combine the two fragments. The combined fragment was digested with two restriction enzymes *Xba*I and *Xho*I, and ligated into the pKOV vector. Afterwards, the resulting plasmid was introduced to the *E. coli* strains and used to delete a target gene by homologous recombination^11^. The plasmids for gene deletion and resultant mutants are listed in Supplementary Table S1.

### Real-time PCR for quantification of mRNA expression levels

The procedure for measuring mRNA level was described before^46^. Briefly, *E. coli* was grown in modified M9 minimal medium until reaching the exponential phase. Cells were harvested in RNase-free vials and centrifuged for 10,000 rpm for 10 min to obtain the cell pellet. Total RNA was extracted using an RNA isolation kit (Macherey–Nagel, Germany). First-strand cDNA was synthesized using a SuperScript III first-strand cDNA synthesis system (Invitrogen, USA). RT-PCR was performed by the StepOne real-time PCR system (Applied Biosystems, USA). The thermal cycling conditions were as follows: denaturation, 1 cycle of 95 °C for 30 s; and amplification, 40 cycles of 95 °C for 15 s, 60 °C for 30 s, and 72 °C for 30 s. The relative mRNA level was calculated using the ΔC_t_ method. The housekeeping gene *rpoD* encoding sigma factor 70 was used as a reference.

### Overexpression of *yohJK* operon

The *yohJK* operon of *E. coli* W (GenBank: *yohJ*, ADT75777.1, and *yohK*, ADT75778.1) was cloned into a low-copy-number plasmid pACYC under the tetracycline-inducible promoter. The sequences of the tetracycline promoter and *yohJK* operon were amplified using PCR, and overlapping PCR for combination of the two fragments followed. Afterwards, the overlapped fragment was digested using *Eco*RI and *Bam*HI restriction enzymes and ligated with linearized pACYC to obtain the plasmid pACYC-Ptet-*yohJK*. The resultant plasmid was then introduced into *E. coli* host strains for further experimentation. Kanamycin (Km) at 50 mg/L was added to the culture medium for plasmid maintenance, and 200 ng/mL of anhydrotetracycline was used for induction of *yohJK*.

### Development of GFP-based 3-HP biosensor for comparison of intracellular 3-HP in *E. coli* W strains

Briefly, we modified the 3-HP biosensor previously developed for *P. denitrificans* ATCC 13867 (designated as BS-P) and applied it for *E. coli* W^39^. The 3-HP inducible promoter (*P*_*mmsA*_) and 10 amino acids of its downstream gene (*mmsA*) from *P. denitrificans* ATCC 13867 was fused with *gfp* reporter gene. The *mmsR* gene, encoding for positive transcription factor for *P*_*mmsA*_ promoter, was expressed under the control of IPTG inducible promoter T5. All the DNA fragments were assembled using overlapping PCR to make a *T5-mmsR_P*_*mmsA*_*-gfp* cassette. The obtained cassette was digested using *Bam*HI *and Hind*III restriction enzymes and ligated with linearized pQE80L plasmid (Supplementary Fig. S11). The resulted biosensor module was named as BS-E. Under the control of 3-HP inducible promoter *P*_*mmsA*_, the expression of GFP is dependent on the levels of intracellular 3-HP.

### Whole-cell fluorescence assay

In order to compare the level of intracellular 3-HP between *E. coli* W strains, the recombinant strains harboring the plasmid pQE80L_T5-*mmsR*_*P*_*mmsA*_-*gfp* were inoculated in the modified M9 minimal medium. The inducers such as IPTG and 3-HP were added into the culture medium. Samples were taken at 6-h after inoculation for fluorescence and OD_600_ measurement. The protocol for GFP measurement was described somewhere else^47^. Synergy H1 microplate reader (BioTek instruments, USA) using 486-nm excitation and 535-nm emission filters was used to measure both fluorescence and OD_600_. The measured fluorescence values were normalized to OD_600_ to obtain specific fluorescence (AU/ OD_600_).

## Supplementary information

Supplementary Information.

## Data Availability

The dataset of RNA-seq and ChIP-exo has been deposited to GEO under the accession number GSE129317 (https://www.ncbi.nlm.nih.gov/geo/query/acc.cgi?acc=GSE111095, the secure token to allow a review of record GSE129317 is qdqlyygkvvgzxaf).
