# Supplementary material for: Systems evaluation reveals novel transporter YohJK renders 3-hydroxypropionate tolerance in Escherichia coli
Source: Sci Rep. 2020 Nov 4;10:19064. doi: 10.1038/s41598-020-76120-3 (PMC7642389; doi:10.1038/s41598-020-76120-3)
Supplement: Supplementary file 1 — Supplementary Information. [file 41598_2020_76120_MOESM1_ESM.docx]

**Systems evaluation reveals novel transporter YohJK renders 3-hydroxypropionate tolerance in *Escherichia coli***

Thuan Phu Nguyen-Vo^1,2,3^, Seyoung Ko^1,3^, Huichang Ryu^1^, Jung Rae Kim^2^, Donghyuk Kim^1,*^ and Sunghoon Park^1,2,*^

^1^School of Energy and Chemical Engineering, UNIST, Ulsan 44919, Republic of Korea

^2^School of Chemical and Biomolecular Engineering, Pusan National University, Busan 46241, Republic of Korea

^3^These authors contributed equally to this work.

^*^Co-corresponding authors:

Donghyuk Kim, School of Energy and Chemical Engineering, UNIST, Ulsan 44919, Korea Phone: +82-52-217-2945. Fax: +82-52-217-2309. E-mail: dkim@unist.ac.kr

Sunghoon Park, School of Energy and Chemical Engineering, UNIST, Ulsan 44919, Korea Phone: +82-52-217-2565. Fax: +82-52-217-2309. E-mail: parksh@unist.ac.kr

**Supplementary information**

*Supplementary Figures*


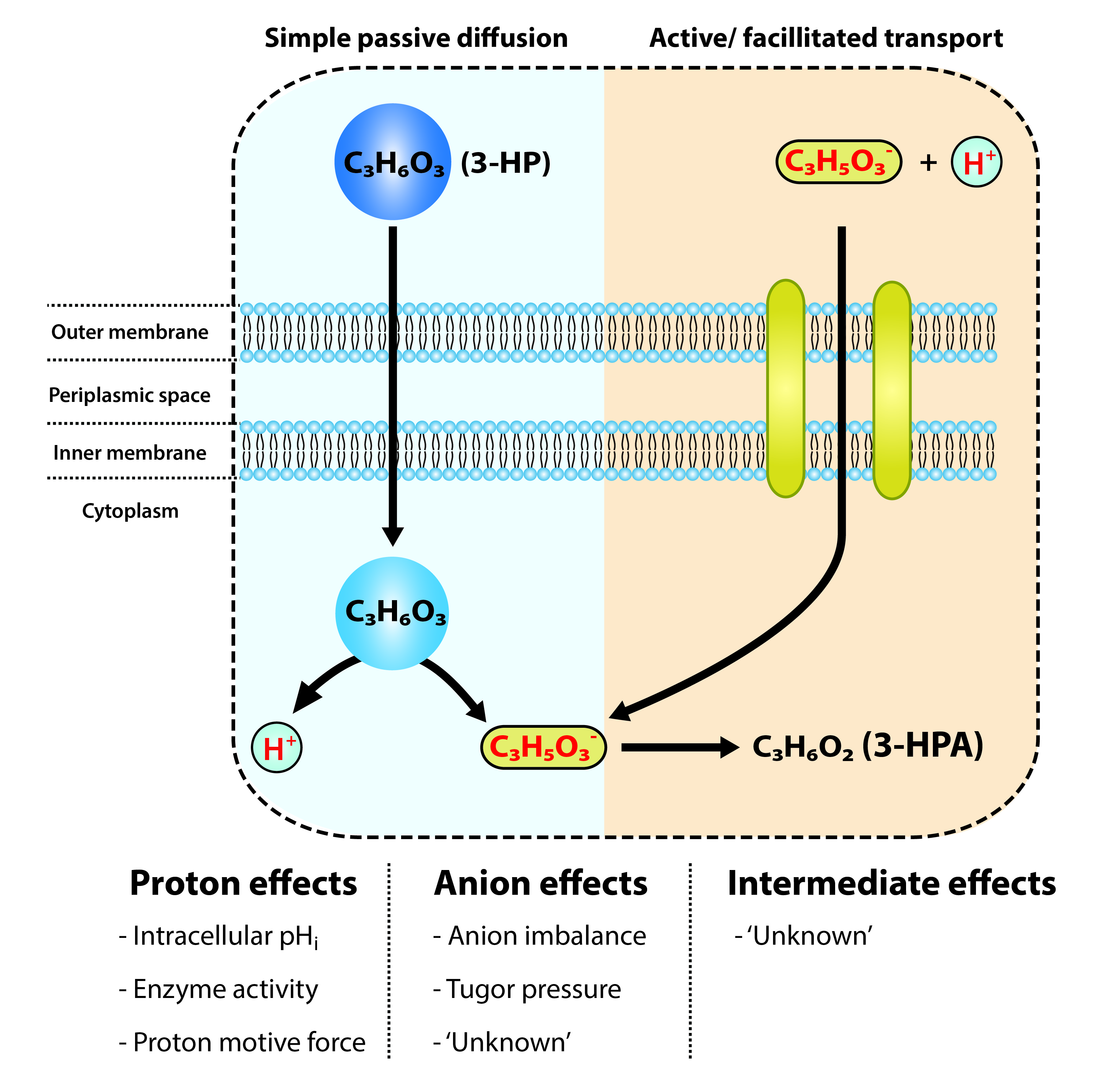


**Figure S1.** **Postulated toxic effects of 3-HP.** 3-HP is expected to enter the cell by simple passive diffusion and/or active/facilitated transport. The simple passive diffusion is possible for undissociated form only. Once entering the cells 3-HP dissociates into proton and anion, and disrupts cellular physiological function by several effects categorized as proton effects, anion effects, and (catabolic) intermediates effects. ‘Unknown’ toxic effects include inhibition to essential enzymes and generation of toxic catabolic intermediates such as 3-hydroxypropionaldehyde (3-HPA).

**
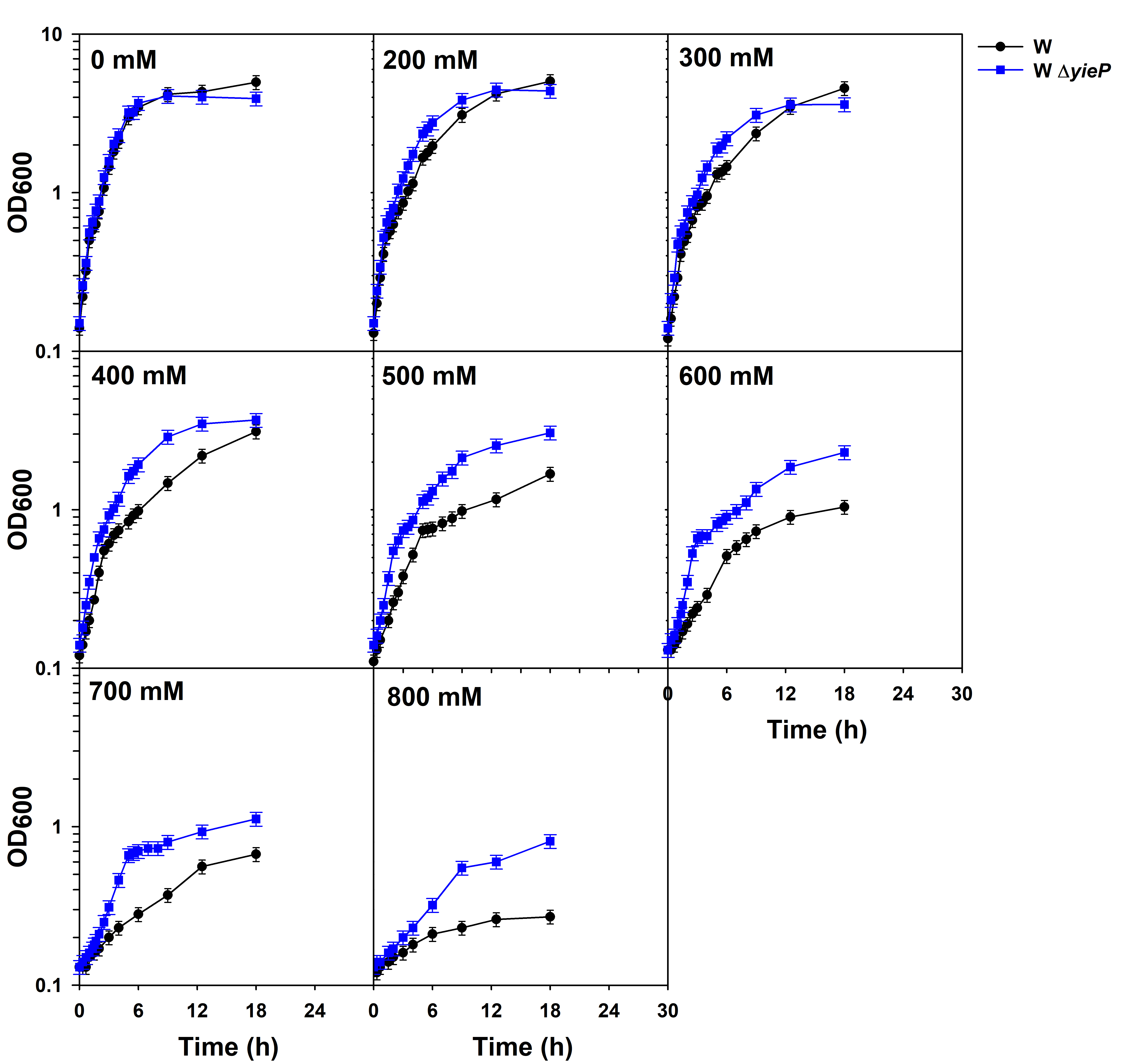
**

**Figure S2.** **Effect of yeast extract on improvement of 3-HP tolerance of *E. coli* W and W Δ*yieP***. *E. coli* W strains were grown in the modified M9 minimal medium with supplementation of 0.5 g/L yeast extract. 3-HP in the range of 0 to 800 mM was added to the culture medium. Symbols: *E. coli* W (blue rectangles), *E. coli* W Δ*yieP* (black circles)

**

**

**Figure S3. Genome-wide RNA-seq analysis of *Escherichia coli* W strains**. (**A**) Number of differentially expressed genes in four groups. Symbols: WΔ and W represent the mutant *E. coli* W Δ*yieP* and wild-type *E. coli* W, respectively; ‘+’ and ‘-’ in the parentheses indicate the presence and absence of 100 mM of 3-HP in the culture medium. (**B**) Hierarchical clustering of differentially expressed genes from the RNA-seq.

**

**

**Figure S4. COG analysis for W(+)/W(-) and W**Δ**(+)/W**Δ**(-).**


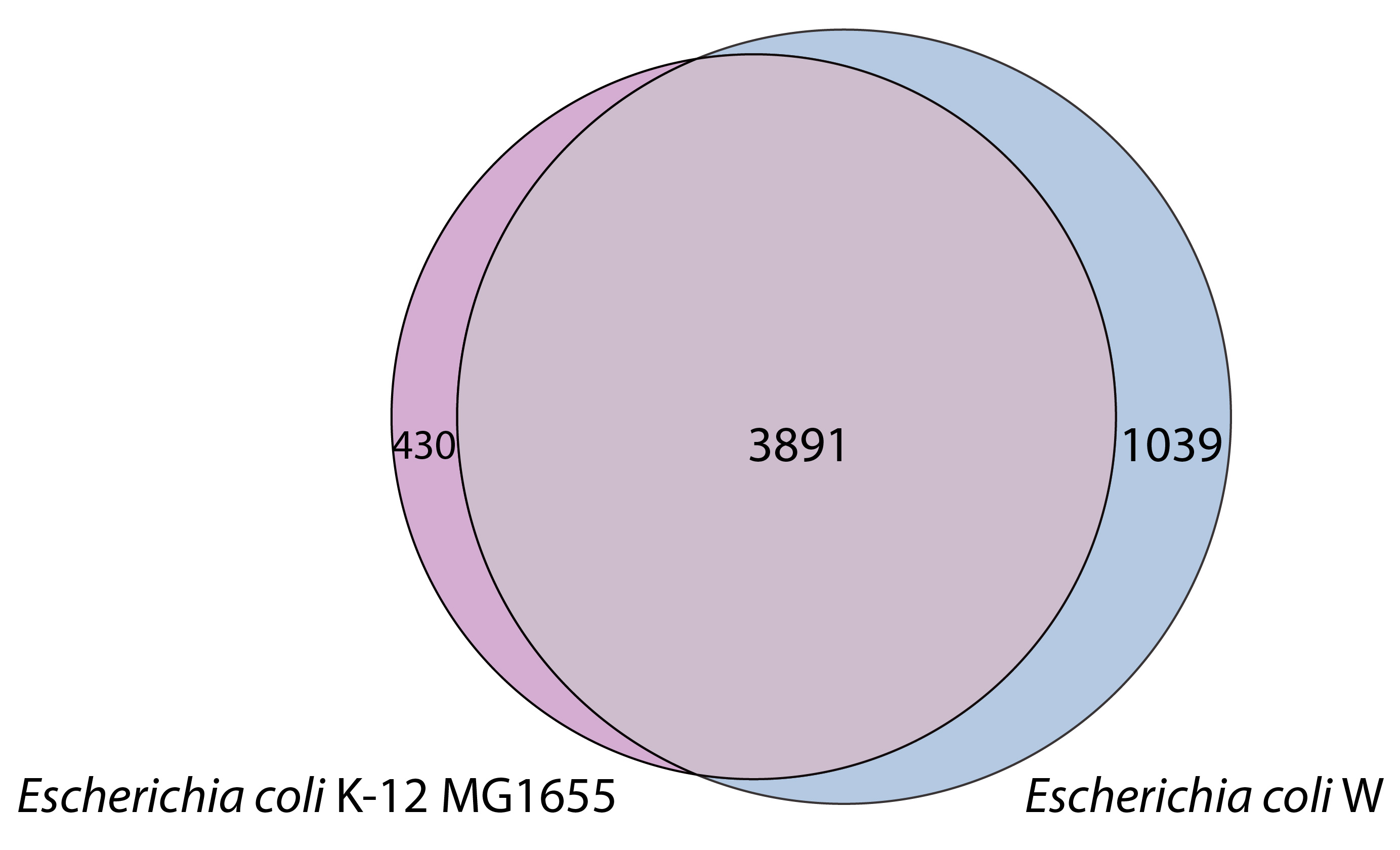


**Figure S5. Comparison of genomes between *E. coli* K-12 MG1655 and *E. coli* W.**

**
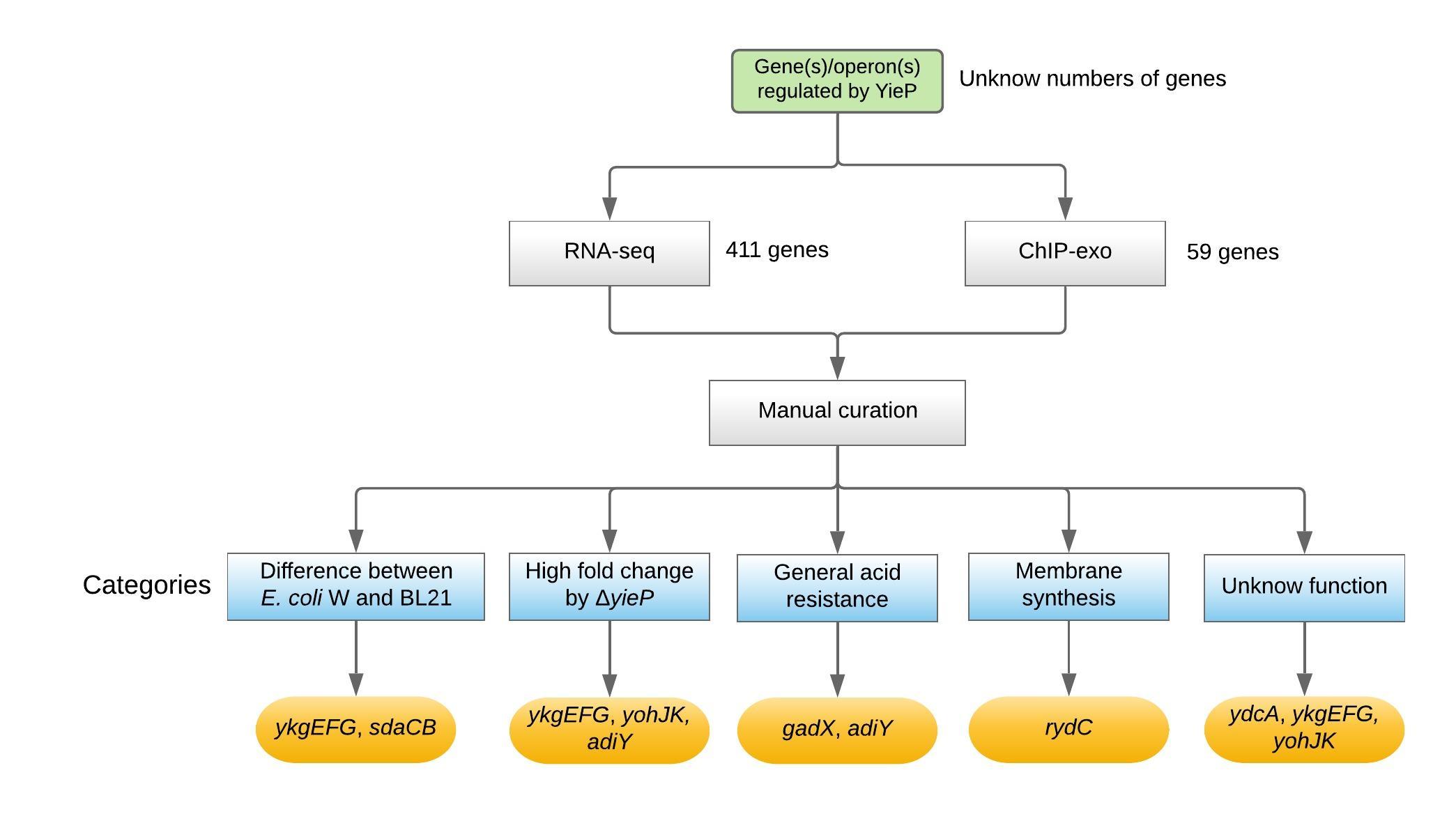
**

**Figure S6. Identification and selection of target genes for functional studies**. Five categories were looked into for manual curation of the genes/operons suggested by the genome-scale RNA-seq and ChIP-exo analyses.


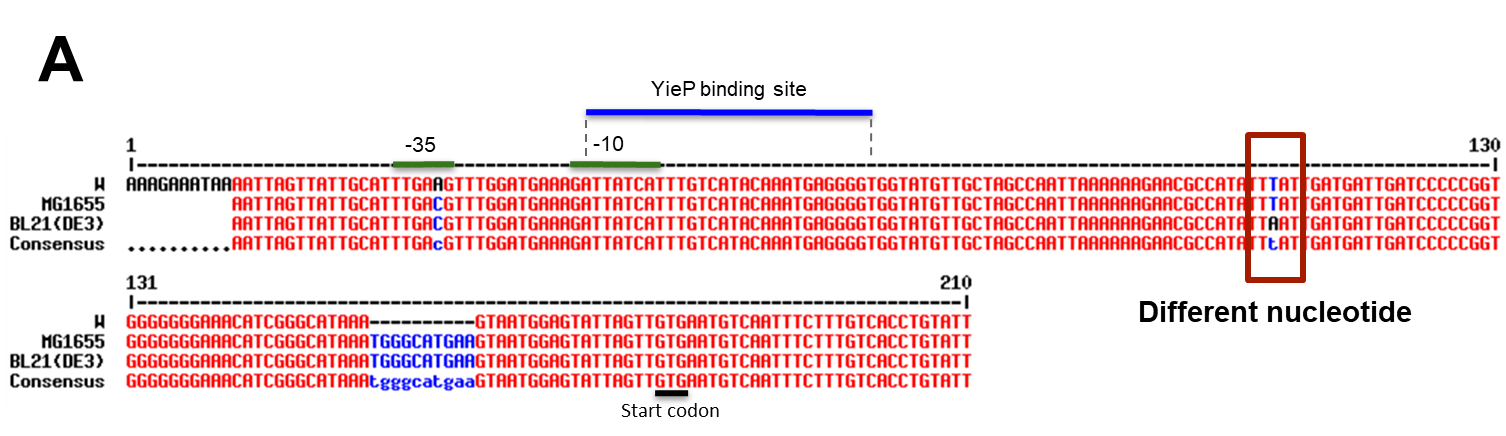


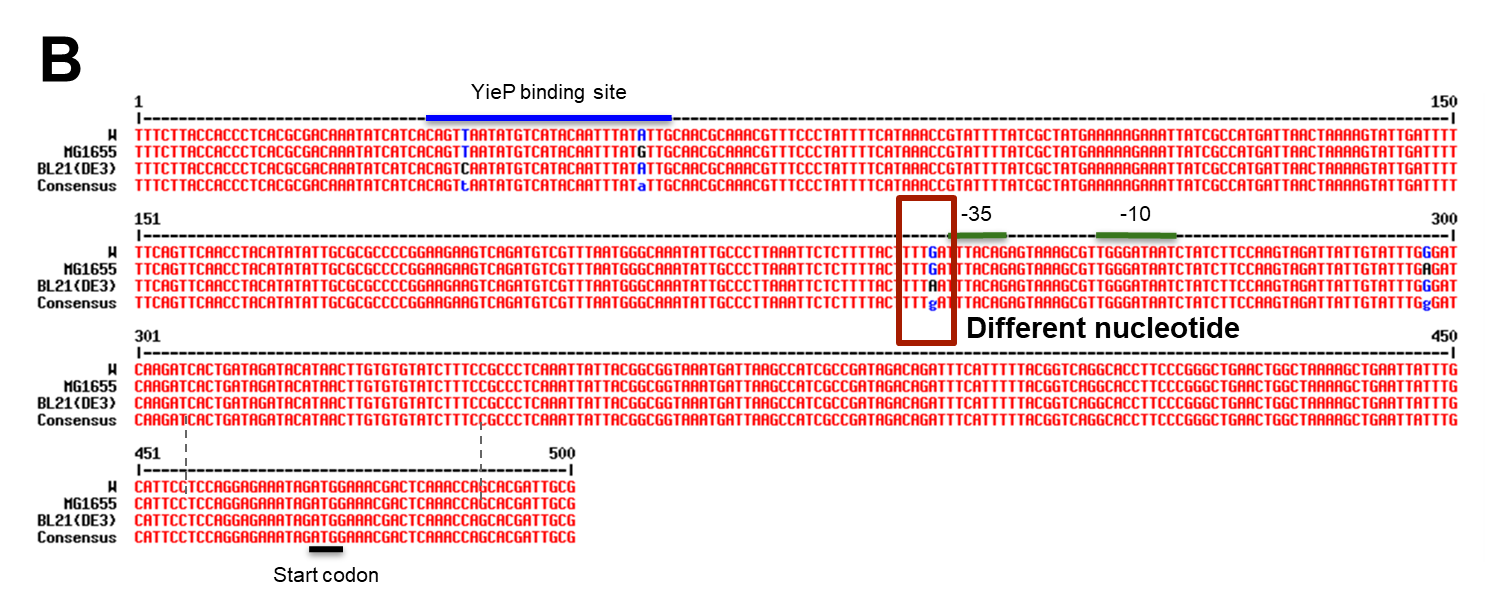


**Figure S7. The difference in promoter sequences between *E. coli* W (or K-12 MG1655) and *E. coli* BL21(DE3).** Promoter sequences of *ykgEFG* and *sdaCB* operon were compared among three *E. coli* strains, W, K-12 MG1655 and BL21(DE3). Different nucleotides were indicated in the red box. YieP binding sites were indicated by blue lines. **(A)** and **(B)** represent the promoter sequences of *ykgEFG* and *sdaCB*, respectively.

**
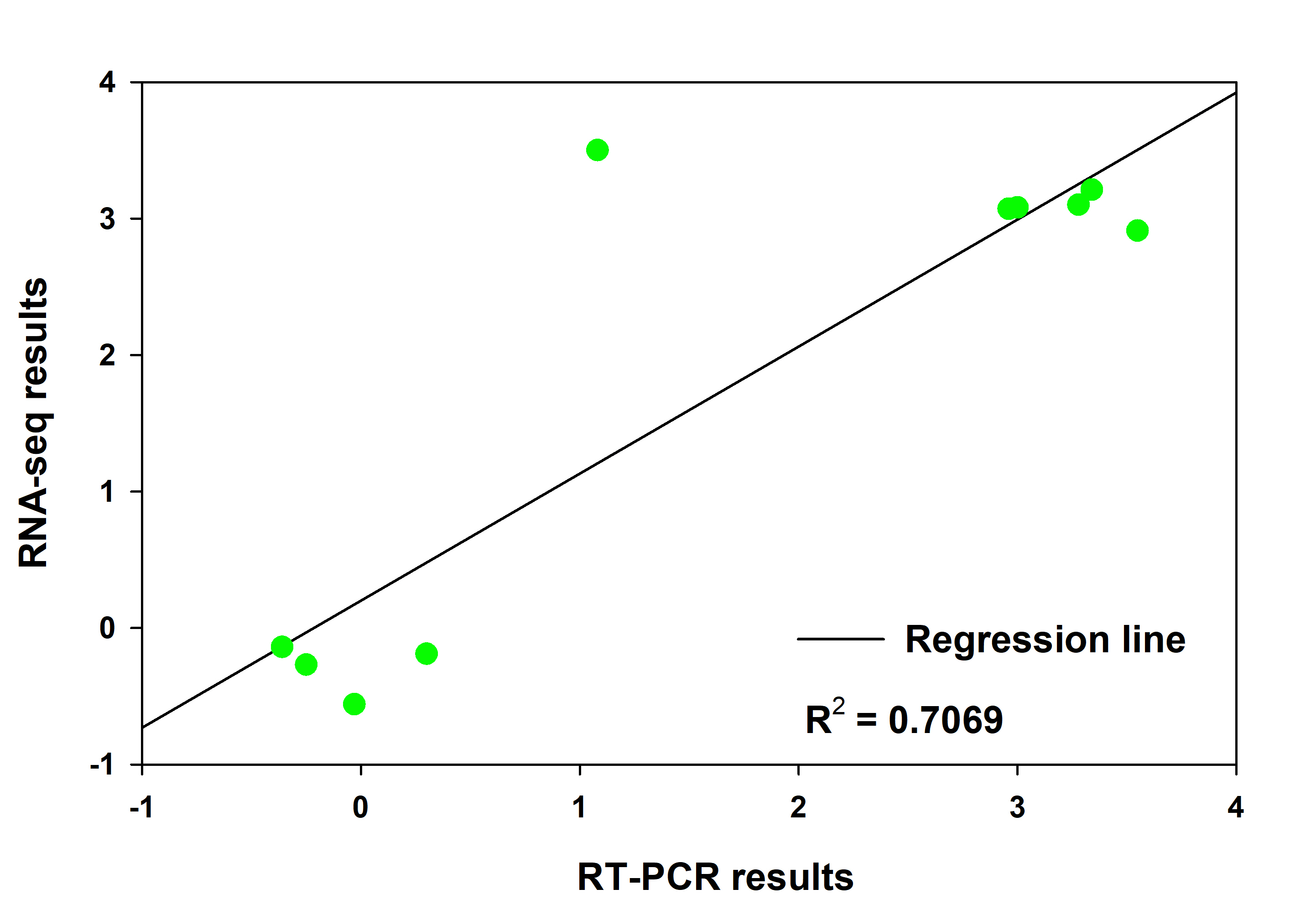
**

**Figure S8.** **Comparison of RNA-seq and RT-PCR measurement for selected genes/operons (*ykgEFG*, *yohJK*, *adiY*, *gadX*, *ydcA*, *sdaCB*)**. The log_2_(fold change) values, where fold change means the ratio of gene expression in W Δ*yieP* to that in W, were indicated. The correlation coefficient of two measurements (R^2^) was ~0.71.





**Figure S9.** **Regulation of *ydcA* by pH response transcription factor *ydcI*.**

**
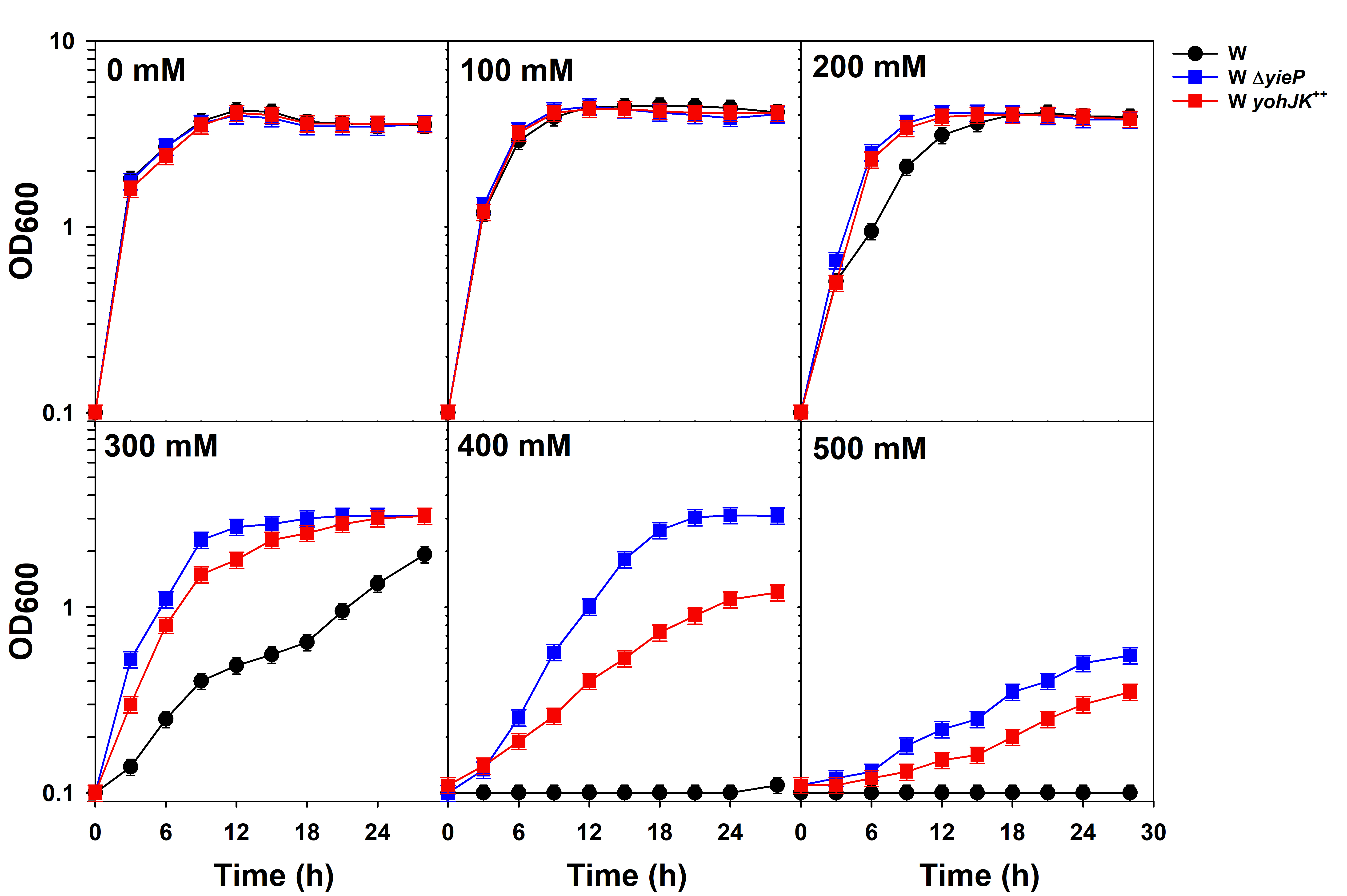
**

**Figure S10.** **Tolerance of *yohJK* overexpressed strain (*yohJK*^++^) against 3-HP**. Three recombinant strains, *E. coli* W and W Δ*yieP* harboring pACYC empty plasmid, *E. coli* W *yohJK*^++^ overexpressing *yohJK* in pACYC, were growth in modified M9 minimal medium supplemented with varying concentrations of 3-HP (0 – 500 mM). Symbols: W (black circles), W Δ*yieP* (blue rectangles), W *yohJK*^++^ (red rectangles).

**
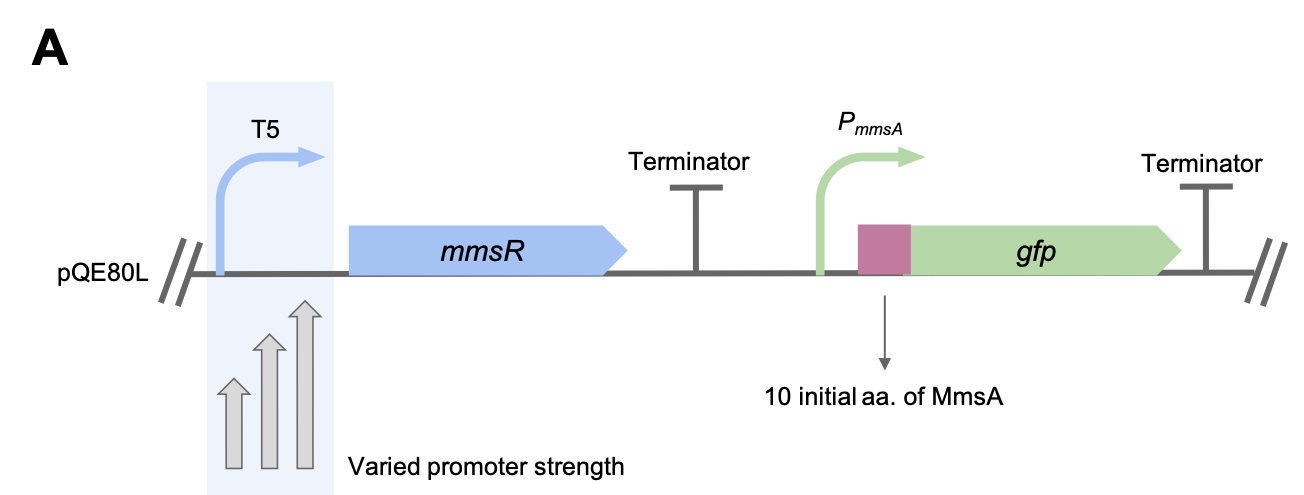
**

**
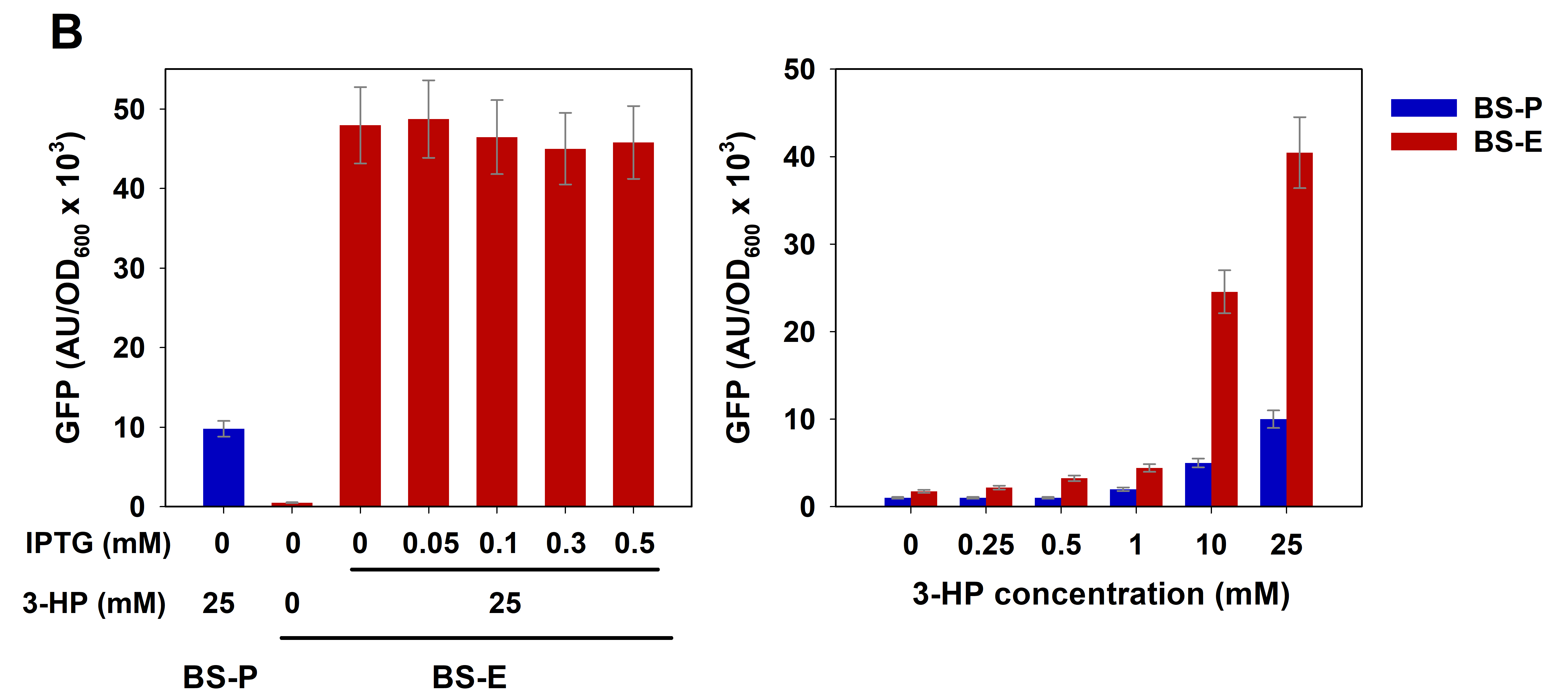
**

**Figure S11.** **Development of GFP-based biosensor for sensing intracellular 3-HP in *E. coli***. **(A)** Construction of biosensor in pQE80L plasmid. Transcription factor *mmsR* was expressed under the control of IPTG inducible promoter T5, 3-HP inducible promoter *P_mmsA_* and 10 amino acids of downstream gene (*mmsA*) was fused with *gfp*. Rho-independent terminators was cloned right after each gene. **(B)** Performance of new biosensor (BS-E) compared with previously developed biosensor (BS-P). Recombinant strains harboring biosensors were cultured in modified M9 minimal medium. GFP was measured at 3 hours after inoculation. Left-hand side: IPTG concentration was varied from 0 – 0.5 mM; right-hand side: IPTG concentration was 0.1 mM, 3-HP concentration was varied from 0 – 25 mM. Symbols: previously developed biosensor, BS-P (blue bar); newly developed biosensor in this study, BS-E (red bar).


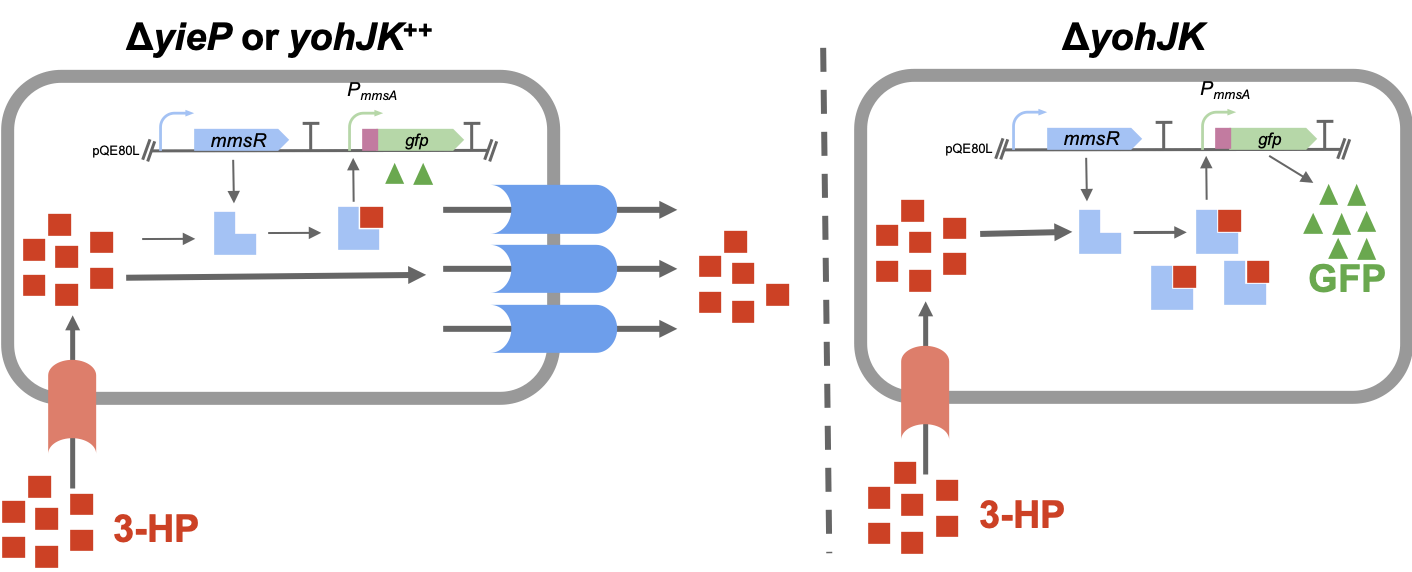


**Figure S12. Working principle of GFP-biosensor and the effect of *yohJK* on 3-HP transport.** When 3-HP (red rectangular) is present, MmsR (blue L-shape) binds to 3-HP, activates the 3-HP inducible promoter (*P_mmsA_*), and promotes GFP expression (green triangle). **(Left**) When *yieP* is deleted or *yohJK* is overexpressed, the intracellular 3-HP level reduces due active secretion of 3-HP (blue block and arrows); consequently, less GFP is expressed. (**Right**) Without 3-HP exporter, intracellular 3-HP level is high and so is GFP expression.

*Supplementary Tables*

**Supplementary Table S1**. Strains and plasmids used in this study

| **Strains and**  **plasmids** | **Description** | **Source** |
| --- | --- | --- |
| ***E. coli* strains** |  |  |
| W | *E. coli* W (ATCC 9637) | KCTC, Korea |
| W Δ*yieP* | *E.* W Δ*yieP* | Nguyen-Vo *et al.*, 2019 |
| BL21 (DE3) | *E. coli* BL21 (DE3) | Nguyen-Vo *et al.*, 2019 |
| BL21 (DE3) Δ*yieP* | *E. coli* BL21 (DE3) Δ*yieP* | Nguyen-Vo *et al.*, 2019 |
| K-12 MG1655 | *E. coli* K-12 MG1655 | Nguyen-Vo *et al.*, 2019 |
| K-12 MG1655 Δ*yieP* | *E. coli* K-12 MG1655 Δ*yieP* | Nguyen-Vo *et al.*, 2019 |
| K-12 MG1655 *yieP-8myc* | 8 *myc* tags were fused to *yieP* | This study |
| W Δ*rydC* | *E. coli* W Δ*rydC* | This study |
| W Δ*ydcA* | *E. coli* W Δ*ydcA* | This study |
| W Δ*gadX* | *E. coli* W Δ*rydC* | This study |
| W Δ*adiY* | *E. coli* W Δ*adiY* | This study |
| W Δ*ykgEFG* | *E. coli* W Δ*ykgEFG* | This study |
| W Δ*yohJK* | *E. coli* W Δ*yohJK* | This study |
| W Δ*yieP* Δ*rydC* | *E. coli* W Δ*yieP* Δ*rydC* | This study |
| W Δ*yieP* Δ*ydcA* | *E. coli* W Δ*yieP* Δ*ydcA* | This study |
| W Δ*yieP* Δ*gadX* | *E. coli* W Δ*yieP* Δ*rydC* | This study |
| W Δ*yieP* Δ*adiY* | *E. coli* W Δ*yieP* Δ*adiY* | This study |
| W Δ*yieP* Δ*ykgEFG* | *E. coli* W Δ*yieP* Δ*ykgEFG* | This study |
| W Δ*yieP* Δ*yohJK* | *E. coli* W Δ*yieP* Δ*yohJK* | This study |
| W *yohJK*^++^ | *E. coli* W / pACYC-*P_tet_*-*yohJK* | This study |
| W Δ*yohJK yohJK*^++^ | *E. coli* W Δ*yohJK* /  pACYC-*P_tet_*-*yohJK* | This study |
| W Δ*yieP* Δ*yohJK yohJK*^++^ | *E. coli* W Δ*yieP* Δ*yohJK* /  pACYC-*P_tet_*-*yohJK* | This study |
| BL21 *yohJK*^++^ | *E. coli* BL21 (DE3) / pACYC-*P_tet_*-*yohJK* | This study |
| BS-E | *E. coli* W pQE80L_T5-*mmsR*_*PmmsA*-*gfp* | This study |
| BS-P | *E. coli* W pUCPK_*P_zwf_*-*mmsR*_*PmmsA*-*gfp* | This study |
| **Plasmids** |  |  |
| pKOV_ Δ*rydC* | plasmid used for deletion of *rydC* | This study |
| pKOV_ Δ*ydcA* | plasmid used for deletion of *ydcA* | This study |
| pKOV_ Δ*gadX* | plasmid used for deletion of *gadX* | This study |
| pKOV_ Δ*adiY* | plasmid used for deletion of *adiY* | This study |
| pKOV_ Δ*ykgEFG* | plasmid used for deletion of *ykgEFG* | This study |
| pKOV_ Δ*yohJK* | plasmid used for making *yohJK* | This study |
| pACYC-*P_tet_*-*yohJK* | plasmid used for overexpression of *yohJK* | Nguyen-Vo *et al.*, 2019 |
| pQE80L_T5-*mmsR*_*PmmsA*-*gfp* | Newly developed biosensor for *E. coli* | This study |
| pUCPK_*P_zwf_*-*mmsR*_*PmmsA*-*gfp* | Previously developed biosensor for *P. denitrificans* | Nguyen *et al.*, 2019 |
